# Activation of cannabinoid receptor 2 attenuates Angiotensin II-induced atrial fibrillation *via* a potential NOX/CaMKII mechanism

**DOI:** 10.3389/fcvm.2022.968014

**Published:** 2022-10-14

**Authors:** Dengyue Xu, Chennian Xu, Xiaodong Xue, Yinli Xu, Jikai Zhao, Tao Huang, Zhishang Wang, Qiusheng Zhao, Zijun Zhou, Yuting Huang, Liming Yu, Huishan Wang

**Affiliations:** ^1^Department of Cardiovascular Surgery, General Hospital of Northern Theater Command, Shenyang, Liaoning, China; ^2^Postgraduate College, China Medical University, Shenyang, Liaoning, China

**Keywords:** atrial fibrillation, endocannabinoid system, cannabinoid receptor 2, nicotinamide adenine dinucleotide phosphate oxidase, Ca2+/calmodulin-dependent protein kinase II, oxidative stress

## Abstract

**Background:**

Atrial fibrillation (AF) is the most frequent arrythmia managed in clinical practice. Several mechanisms have been proposed to contribute to the occurrence and persistence of AF, in which oxidative stress plays a non-negligible role. The endocannabinoid system (ECS) is involved in a variety physiological and pathological processes. Cannabinoid receptor 1 (CB1R) and cannabinoid receptor 2 (CB2R) are expressed in the heart, and studies have shown that activating CB2R has a protective effect on the myocardium. However, the role of CB2R in AF is unknown.

**Materials and methods:**

Angiotensin II (Ang II)-infused mice were treated with the CB2R agonist AM1241 intraperitoneally for 21 days. Atrial structural remodeling, AF inducibility, electrical transmission, oxidative stress and fibrosis were measured in mice.

**Results:**

The susceptibility to AF and the level of oxidative stress were increased significantly in Ang II-infused mice. In addition, nicotinamide adenine dinucleotide phosphate oxidase 2 (NOX2), NOX4, and oxidized Ca^2+^/calmodulin-dependent protein kinase II (ox-CaMKII) were highly expressed. More importantly, treatment with AM1241 activated CB2R, resulting in a protective effect.

**Conclusion:**

The present study demonstrates that pharmacological activation of CB2R exerts a protective effect against AF *via* a potential NOX/CaMKII mechanism. CB2R is a potential therapeutic target for AF.

## Introduction

Atrial fibrillation (AF), the most common arrhythmia in clinical practice, affects nearly 2% of the general population and is associated with a pronounced increase in the incidence of heart disease and death (1). During recent decades, our understanding of the mechanisms driving the pathogenesis and maintenance of AF has increased considerably. Atrial remodeling, including electrophysiological changes and structural remodeling, is the main cause of the occurrence and persistence of AF (2). Oxidative stress has been investigated as a potential essential link between AF and atrial remodeling (3). Oxidative stress is an imbalance between oxidants and antioxidants in favor of the former, resulting in excess reactive oxygen species (ROS) accumulation and leading to cellular damage. Numerous studies have shown that excess ROS directly affect ion channels and cause electrical remodeling (4). In addition, research evidence suggests that hydroxyl radicals, a type of ROS, can alter myofibrillar protein structure and function and further contribute to structural remodeling, which is a feature of atrial fibrosis (5). Given this, reducing oxidative stress may be an attractive target for the treatment of AF.

The endocannabinoid system (ECS) consists of endogenous ligands, enzymes for synthesis, degradation and transport, and cannabinoid receptors (CBRs). Cannabinoid receptor 1 (CB1R) was cloned from a rat cerebral cortex cDNA library in 1987 and characterized as the specific membrane receptor of a cannabinoid compound 1 year later. It was not until 5 years later that another cannabinoid receptor was found in macrophages of the spleen and named cannabinoid receptor 2 (CB2R). The endogenous ligands anandamide (AEA) and 2-arachidonoylglycerol (2-AG) were discovered in 1992 and 1995 (6). The ECS has been reported to play an important role in the pathogenesis and progression of diseases (7), and pharmacological inhibition of CB1R or activation of CB2R exerts cardioprotective effects (8). Unlike CB1R, which is mainly distributed in the central nervous system, CB2R has gained attention as an important therapeutic target due to its lack of psychoactivity. Moreover, it has been confirmed in various animal models of atherosclerosis and myocardial ischemia/reperfusion injury that CB2R activation by natural and synthetic agonists shows antioxidative effects (9). However, little is known about the role of CB2R in AF, and we were curious as to whether it can decrease oxidative stress in AF.

Mitochondria, dynamic organelles regulated by fission and fusion, are major sources of ROS (10). Nicotinamide adenine dinucleotide phosphate (NADPH) oxidase (NOX) is the most important enzyme in oxidative stress, and it has been reported that Ca^2+^/calmodulin-dependent protein kinase II (CaMKII), a well-described serine-threonine kinase related to cardiovascular diseases (11), can be oxidized by NOX at methionines 281 and 282 to form oxidized CaMKII (ox-CaMKII) (12). In addition, a series of studies have confirmed that ox-CaMKII plays a proarrhythmic role in both AF and sinoatrial node dysfunction and functions upstream of ROS by inducing mitochondrial damage (13–16).

Therefore, the CB2R agonist AM1241 was used as an adjuvant to reduce the susceptibility of AF in Angiotensin II (Ang II)-infused mice. In addition, the involvement of the NOX/CaMKII mechanism in the protective action of AM1241 was evaluated.

## Materials and methods

### Animals and treatment

Eight-week-old male C57BL/6 mice (HUAFUKANG Bioscience Co., Ltd., Beijing, China) were kept under standard housing conditions. After a 1-week acclimatization period, the mice were randomly assigned to 5 groups (*n* = 15 for each group): the control group, the Ang II group, the Ang II + vehicle (Veh) group, the Ang II + AM1241 group, and the Ang II + AM630 group. Ang II (2,000 ng/kg/min) (A1042, APExBIO, Houston, TX, USA) was subcutaneously infused into the mice for 3 weeks with osmotic pumps (Alzet model 2004; Durect, CA, USA). The control group underwent sham operation. The CB2R agonist AM1241 (S1544, Selleck Chemicals, Houston, TX, USA) and CB2R antagonist AM630 (HY-15421, MedChemExpress, Monmouth Junction, NJ, USA) were dissolved in solvents containing DMSO, Tween-20 and PBS (1:1:8). The concentration of AM1241 was 20 mg/kg, the concentration of AM630 was 3 mg/kg, and the solvent was injected into the Ang II + Veh group (8). The drug injection volume per day was 0.1 mL per mouse for 21 days. For the first time, the drugs were administered intraperitoneally for 1 h before the pumps were implanted, and then the injection was performed at the same time every day.

### Blood pressure measurement and echocardiographic measurement of the left atrium

The blood pressure (BP) of mice was measured before and at the end of treatment with a tail-cuff system (BP-2010, Softron Biotechnology, Beijing, China). Each measurement was repeated three times, and the average BP was calculated. On the 21st day post-Ang-II infusion, echocardiography was performed with a high-resolution ultrasound imaging system (D700, Vinno, Suzhou, China). Mice were anesthetized with 1.5% isoflurane. The left atrium (LA) movement was recorded on the parasternal long-axis view, and the left atrium diameter (LAD) was measured at the left ventricular end systole.

### Atrial fibrillation stimulation

Surface electrocardiogram (ECG) was recorded in anesthetized mice (1% pentobarbital sodium) using PowerLab monitoring systems and LabChart v7 software (ADInstruments, Castle Hill, Australia). A 1.1 F octapolar electrode catheter (Transonic Scisense Inc., Ontario, Canada) was inserted into the right atrium *via* the jugular vein. The inducibility of AF was evaluated by bust stimulation (iWorx System Inc., Dover, NH, USA) for 2 s starting with a cycle length (CL) of 40 ms and decreasing in each burst by a 2 ms decrement to a CL of 20 ms (17). The burst pacing protocols were repeated three times after stabilization for 5 min, and AF was considered to be inducible if 2 or 3 bursts evoked an AF episode for at least 2 s on the ECG. AF duration was defined as the total period of time from the end of burst pacing to the first P wave detected in each individual mouse (18).

### Atrial electrophysiological mapping

Atrial electrophysiological mapping was performed according to a previous study (19). In brief, the mice were placed on the operating table after anesthetizing and ventilation (Taimeng Technology, Chengdu, China). The thorax was opened, and the LA was exposed. A 64-electrode microelectrode array was placed on the surface of the LA. A 64-channel electrophysiological mapping system (MappingLab Inc., UK) was used to record electrograms. The mean conduction velocity and absolute inhomogeneity were acquired using EMapScope5 software (MappingLab Inc., UK). Then, the mice were sacrificed immediately for blood and heart samples.

### Serological experiment

The serum samples were separated by centrifugation in anticoagulant tubes (1,000 *g*, 10 min). The activity of superoxide dismutase (SOD) and the contents of malondialdehyde (MDA), glutathione (GSH), Ang II, and aldosterone (ALD) were determined by colorimetric reagent kits from the Nanjing Jiancheng Bioengineering Institute.

### Histological staining analysis

Atrial paraffin sections at 5 μm were deparaffinized and hydrated and then stained by H&E staining (KGA224, KeyGEN Bio TECH Corp., Jiangsu, China) and Masson’s trichrome staining (BA4079, Baso Diagnostics Inc., Zhuhai, China). The pictures were captured by a UB203i microscope (Aopu Technology, Chongqing, China). To visualize the ROS in the atrium, frozen atrial sections at 8 μm were incubated with 20 μM dihydroethidium operating fluid (KGAF019, KeyGEN Bio TECH Corp., Jiangsu, China) at room temperature for 30 min. All fluorescence images were taken by a Nikon C2 Plus confocal microscope (Nikon, Tokyo, Japan). At least 3 fields from each sample were randomly selected for quantitative analysis *via* ImageJ software.

### Transmission electron microscopy

Atrial samples (1 mm^3^ blocks) were fixed with 2.5% glutaraldehyde at 4°C overnight and then postfixed in 1% osmium tetroxide for 1 h. The blocks were dehydrated with a graded series of ethanol solutions and embedded in epoxy resin. Thereafter, they were cut into 60 nm ultrathin sections (EM UC7, Leica, Vienna, Austria), which were subsequently observed with a transmission electron microscope (TEM) (HI7700, Hitachi, Tokyo, Japan). The images were analyzed using ImageJ software.

### Western blot analysis

Total proteins were prepared using RIPA lysis buffer (abs9225, Absin, Shanghai, China) containing 1% protease inhibitor (BP101, Bio-Platform, Shanghai, China). Tissue lysates were separated by electrophoresis and transferred onto polyvinylidene difluoride (PVDF) membranes (IPVH00010, Merck Millipore Ltd., MA, USA) by the sandwich method. The membranes were incubated with primary antibodies against CB2R (PA569179, Thermo Fisher, Shanghai, China), CaMKII (4436, Cell Signaling Technology, Boston, MA, USA), and ox-CaMKII (36254, GeneTex, Southern California, CA, USA) overnight at 4°C followed by a horseradish peroxidase peroxidase-conjugated secondary antibody for 1 h at room temperature. The blots were detected by enhanced chemiluminescence (P10300, NCM, Suzhou, China) and were scanned by a Tanon image analysis system (5200, Tanon, Shanghai, China).

### Statistical analysis

All data are presented as the mean ± SEM (standard error of the mean). Differences were assessed with Student’s *t* test or one-way analysis of variance (ANOVA), followed by Bonferroni *post hoc* test (v9.0, GraphPad Software, San Diego, CA, USA). A value of *P* < 0.05 was considered to indicate statistical significance.

## Results

### Under Angiotensin II stimulation, the RAAS was activated, and blood pressure rose

**FIGURE 1:**
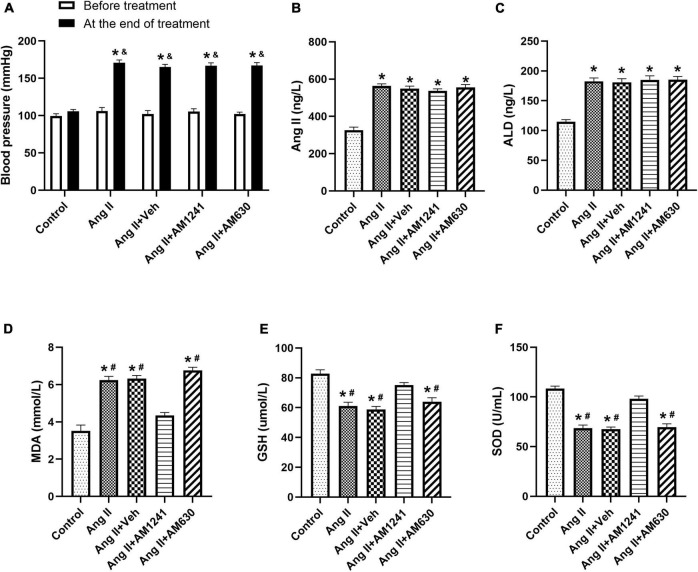
Under Angiotensin II stimulation, the RAAS was activated, and blood pressure (BP) rose. Ang II (2,000 ng/kg/min) was subcutaneously infused into the mice for 21 days. In addition, solvent (0.1 ml), AM1241 (20 mg/kg), and AM630 (3 mg/kg) were administered intraperitoneally every day to the animals. Before the treatment, there were no significant differences in blood pressure (BP) among the five groups. After 21 days of different treatments, BP increased significantly in all groups except the control group **(A)**. The content of Angiotensin II **(B)**, aldosterone (ALD) **(C)**, malondialdehyde (MDA) **(D)** and glutathione (GSH) **(E),** and the enzyme activity of superoxide dismutase (SOD) **(F)** were determined by serological examination. The data represent the mean ± SEM. *P < 0.05 vs. control group. &*p* < 0.05 vs. before treatment. #*P* < 0.05 vs. Ang II + AM1241 group.

Before the treatment, there were no significant differences in BP among the five groups. After 21 days of treatment, there was still no significant change in the control group. However, the BPs of the other four groups rose significantly compared with that of the control group in the same period (Figure 1A). Serological examination showed that the levels of Ang II and ALD in the four groups other than the control group were higher than those before treatment (Figures 1B,C). AM1241 and AM630 did not affect BP or the levels of Ang II and ALD.

### Administration of AM1241 reduced the inducibility of atrial fibrillation in Angiotensin II-infused mice

**FIGURE 2:**
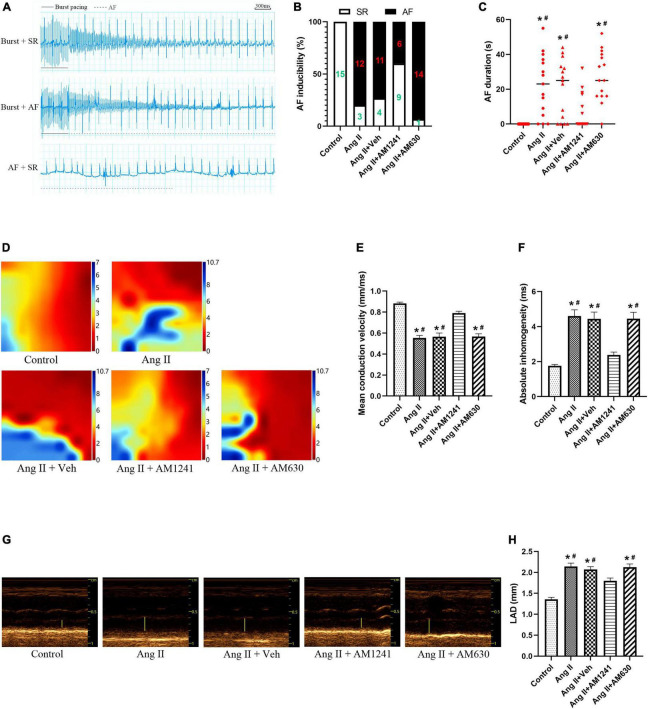
Administration of AM1241 reduced the inducibility of atrial fibrillation (AF) in Angiotensin II-infused mice. **(A)** Simultaneous recordings of surface electrocardiogram (ECG) after 21-day infusion of Ang II (2,000 ng/kg/min). Solid underlines indicate the burst pacing period, and dashed underlines highlight the AF period. The above two ECGs recorded representative ECG waves of AF and SR after a burst of electrical stimuli. The bottom recorded ECG waves of AF followed by spontaneous conversion to SR. Bar = 300 ms. **(B)** Inducibility of AF. **(C)** Duration of AF. **(D)** Electrophysiological mapping images of spontaneous conduction in the LA. The bars from red to blue indicate the total time from the first to the last measurement within one heartbeat. **(E)** Mean conduction velocity. **(F)** Absolute inhomogeneity of left atrial electrical conduction. **(G)** Representative M-mode echocardiography of the LA. **(H)** Quantification of LAD. The data represent the mean ± SEM. *P < 0.05 vs. control group. #*P* < 0.05 vs. Ang II + AM1241 group.

Compared to those in the control mice, the inducibility and duration of AF in Ang II-infused mice were increased significantly. However, after administration of AM1241, the inducibility and duration decreased, while they did not change significantly after AM630 treatment (Figures 2A–C). In addition, the electrophysiological mapping experiment showed that Ang II significantly decreased the mean conduction velocity and increased absolute inhomogeneity. These effects were also suppressed by AM1241 administration, and AM630 treatment did not affect the above changes (Figures 2D–F). Next, the echocardiographic data showed that AM1241 administration attenuated Ang II-induced LA chamber enlargement, and AM630 treatment did not (Figures 2G,H).

### Administration of AM1241 attenuated oxidative stress in the atrial tissue of Angiotensin II-infused mice by activating cannabinoid receptor 2

**FIGURE 3:**
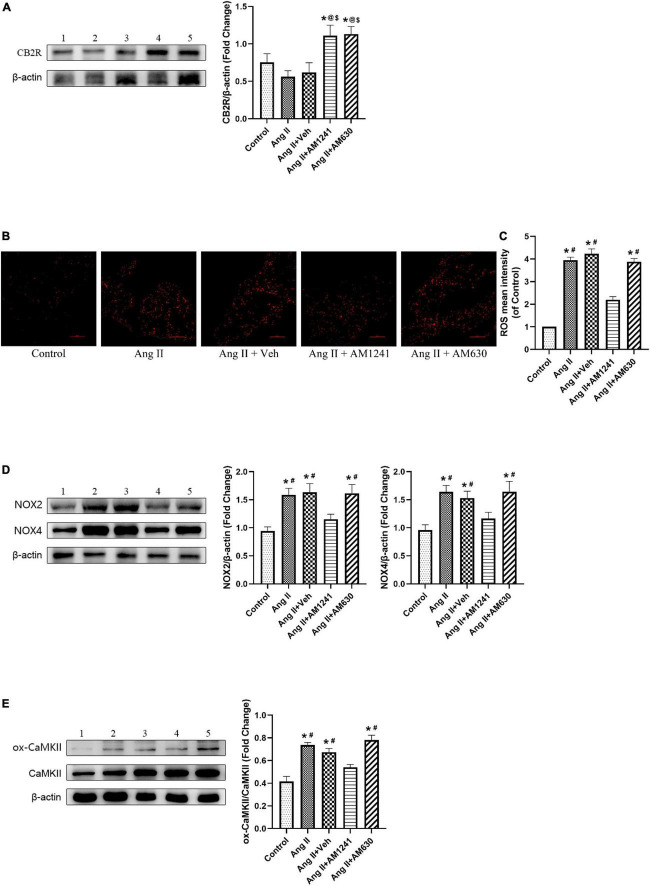
Administration of AM1241 attenuated oxidative stress in the atrial tissue of Angiotensin II-infused mice by activating cannabinoid receptor 2 (CB2R). **(A)** Representative western blot image and relative expression of CB2R. **(B)** Representative picture of reactive oxygen species (ROS) (red) by dihydroethidium staining. Bar = 100 μm. **(C)** Quantitative analysis of reactive oxygen species (ROS) mean intensity. **(D)** Representative western blot image and relative expression of NOX2 and NOX4. **(E)** Representative western blot image and relative expression of ox-CaMKII and CaMKII. Lane 1: control group; Lane 2: Ang II group; Lane 3: Ang II + Veh group; Lane 4: Ang II + AM1241 group; Lane 5: Ang II + AM630 group. The data represent the mean ± SEM. *P < 0.05 vs. control group. #*P* < 0.05 vs. Ang II + AM1241 group. @*P* < 0.05 vs. Ang II group. $*P* < 0.05 vs. Ang II + Veh group.

Immunoblotting of atrial tissue showed that the expression of CB2R was slightly decreased in Ang II-infused mice but increased significantly after administration of AM1241 and AM630 (Figure 3A). MDA is a main lipid peroxidation product that reflects the level of oxidative stress. GSH and SOD are the major antioxidants. Ang II-infused mice showed elevated MDA levels, reduced GSH levels and reduced SOD enzyme activity. The adverse outcomes were remarkably reversed by the addition of AM1241 (Figures 1D–F). Later, oxidative stress in LA was detected. Fluorescence staining demonstrated that the mean ROS intensity was increased in Ang II-infused mice but decreased after treatment with AM1241 (Figures 3B,C). In addition, western blot analysis showed that the levels of ox-CaMKII, NOX2 and NOX4 were increased in the Ang II-infused mice but were decreased by AM1241 treatment (Figures 3D,E). However, no distinct changes occurred after AM630 administration.

### Administration of AM1241 attenuated mitochondrial damage in Angiotensin II-infused mice

**FIGURE 4:**
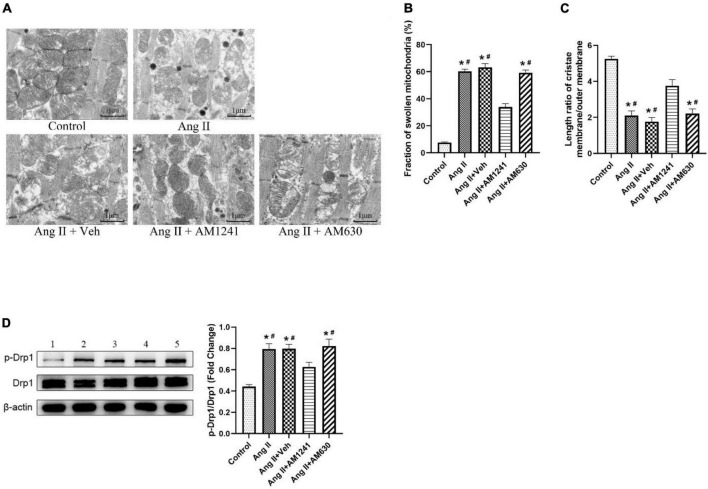
Administration of AM1241 attenuated mitochondrial damage in Angiotensin II-infused mice. **(A)** Representative transmission electron microscopic images of atrial samples. Bar = 1 μm. **(B)** Fraction of swollen mitochondria. **(C)** Length ratio of the mitochondrial cristae membrane and outer membrane, a parameter of mitochondrial cristae density. For each analyzed mitochondrion, the total length of the cristae membrane was obtained by adding the lengths of all the cristae together and multiplying by 2. The ratio was determined by dividing the total length of the cristae membrane by the mitochondrion perimeter. **(D)** Representative western blot image and relative expression of p-Drp1 and Drp1. Lane 1: control group; Lane 2: Ang II group; Lane 3: Ang II + Veh group; Lane 4: Ang II + AM1241 group; Lane 5: Ang II + AM630 group. The data represent the mean ± SEM. *P < 0.05 vs. control group. #*P* < 0.05 vs. Ang II + AM1241 group.

Morphological changes in mitochondria in LA were detected by TEM. As shown in Figure 4A, mitochondria were intact in the control group, and the cristae were in order and clear. After damage by Ang II, most of the mitochondria were swollen and even cavitated, and the cristae were deformed and dissolved. The occurrence of swollen mitochondria increased (Figure 4B), and the length ratio of the cristae membrane to mitochondrial outer membrane decreased significantly (Figure 4C). After AM1241 administration, there were fewer disorganized mitochondria, and the pathological changes were attenuated. Fission is an important feature of mitochondrial dynamics, and phosphorylation of Drp1 Ser616 promotes mitochondrial fission. In Ang II-infused mice, western blotting confirmed the increased phosphorylation of Drp1 at Ser616 (Figure 4D). Notably, AM1241 showed deterrent effects on Drp1 phosphorylation, and there was no significant difference in the above changes between the Ang II + AM360 group and Ang II group.

### Administration of AM1241 attenuated atrial fibrosis in Angiotensin II-infused mice

**FIGURE 5:**
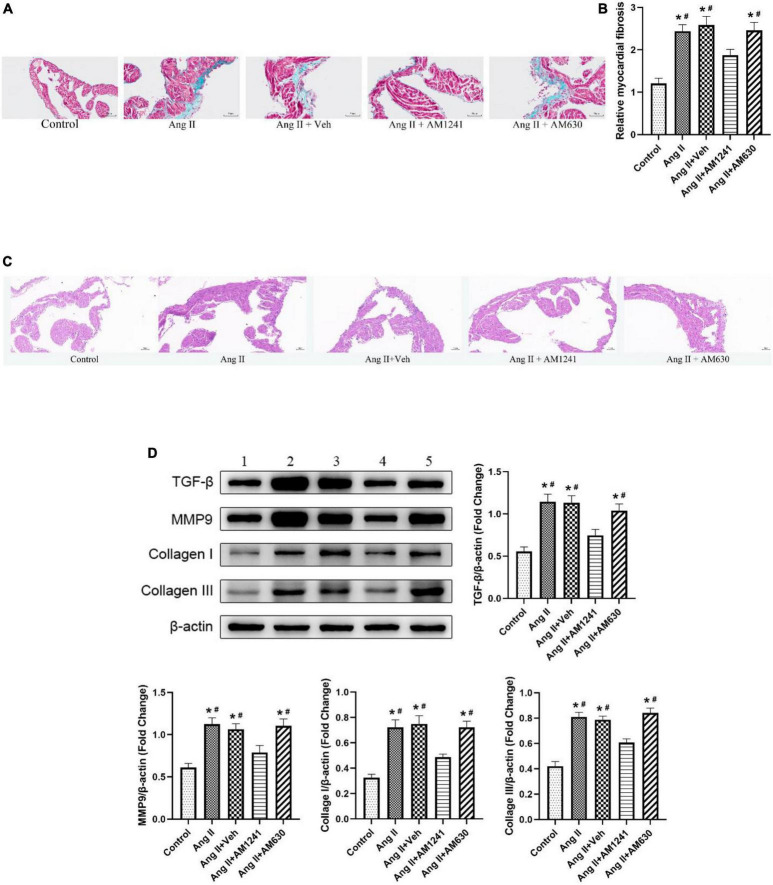
Administration of AM1241 attenuated atrial fibrosis in Ang II-infused mice. **(A)** Representative images of Masson’s trichrome staining. Bar = 50 μm. **(B)** Relative myocardial fibrosis. **(C)** Representative images of H&E staining. Bar = 50 μm. **(D)** Representative western blot image and relative expression of TGF-β, MMP9, collagen I and collagen III. Lane 1: control group; Lane 2: Ang II group; Lane 3: Ang II + Veh group; Lane 4: Ang II + AM1241 group; Lane 5: Ang II + AM630 group. The data represent the mean ± SEM. *P < 0.05 vs. control group. #*P* < 0.05 vs. Ang II + AM1241 group.

Atria from Ang II-infused mice displayed large fibrotic areas, as indicated by Masson’s trichrome staining and H&E staining, while treatment with AM124 alleviated fibrosis (Figures 5A–C). Moreover, significant atrial fibrosis was evidenced by western blotting analysis of TGF-β, MMP, collagen I and collagen III, but the expression of these proteins was restored in mice in the Ang II + AM1241 group (Figure 5D). As with previous results, AM630 did not ameliorate the impact of Ang II.

## Discussion

As the global life expectancy increases, the prevalence of AF in the general population is rising sharply. Thus, AF is becoming an urgent public health issue. In addition, AF may seriously affect patients’ quality of life, as it is related to serious complications, such as stroke, heart failure, and cardiac arrest, which result in increased morbidity, mortality and health care costs (20). Therefore, effective treatment of AF is critically needed. AF treatment is largely based on catheter ablation. The currently available pharmacotherapies for AF have poor efficacy, mainly because they are not directed at the molecular cause of AF (21). Oxidative stress and fibrosis are indispensable pathogenic mechanisms in AF. The experimental results showed that the susceptibility of Ang II-infused mice to AF was significantly increased and that oxidative stress and fibrosis were also altered. Notably, these changes were reversed after treatment with the CB2R receptor-specific agonist AM1241, while the adverse consequences of Ang II were not improved after administration of the CB2R antagonist AM630.

Angiotensin II is the main regulator of cardiac oxidative stress and increases the production of ROS in the cardiovascular system by activating membrane-bound NOX, endoplasmic reticulum stress and mitochondrial oxidative stress (22). ROS can ultimately cause myocardial fibrosis and promote the appearance and persistence of AF. This creates a dangerous positive feedback loop, predisposing patients to AF and thus further fibrosis (23). Moreover, Professor Anderson found that targeted loss of ox-CaMKII in oxidation-resistant CaMKII MMVV mice [a mouse model whose methionine pair (281/282) was replaced by valine] was sufficient to prevent the proarrhythmic response of Ang II in AF (16). They reasoned that CaMKII is an upstream signal of ROS and can be activated by NOX (12). In our study, the expression of NOX2 and NOX4 was increased in the Ang II-infused mice, and ox-CaMKII also changed accordingly, resulting in mitochondrial damage; this enhanced oxidative stress and fibrosis and ultimately increased the susceptibility to AF.

The ECS is an intricate and heterogeneous signaling network composed of (i) endogenous cannabinoids (eCBs) such as AEA and 2-AG and other cannabimimetic ligands, e.g., oleoylethanolamide and stearoylethanolamine; (ii) metabolic enzymes such as fatty acid amide hydrolase (FAAH), monoacylglycerol lipase (MAGL) and other enzymes that regulate eCBs; and (iii) the G protein-coupled receptors CB1R and CB2R (6). The interactions among them controls the activity level of the ECS, which plays a vital role in pathophysiology during each stage of life (24). In the cardiovascular system, the expression of CBRs is not high, and it is still unclear what role the ECS plays in the physiological process, but under pathological conditions, the expression of ECS-related components undergoes dramatic changes. Sugamura et al. found that the levels of AEA and 2-AG in blood samples of patients with coronary heart disease were significantly increased and that the expression of CB1R was increased in coronary atherosclerotic plaques (25). Interestingly, in patients with ischemic cardiomyopathy, the expression of CB1R in myocardial tissue is increased, and the expression of CB2R is decreased (26). Moreover, in mouse models, inhibition of CB1R or activation of CB2R alleviates quetiapine (27) or clozapine-induced (28) myocardial damage. Considering the possible undesirable side effects of CB1R modulators (9), the current experiment was carried out for CB2R. We hypothesized that the expression of CB2R would decrease with continuous Ang II infusion, while pharmaceutical agonism of CB2R would reduce susceptibility to AF. Ultimately, our results fully verified this conjecture.

Cannabinoid receptor 2 (CB2R) activation has an obvious protective effect on the myocardium. It has been confirmed that activation of CB2R reduces necroptosis of the myocardium induced by ethanol (8). It has also been reported that CB2R deficiency impairs regulation of immune activity and apoptosis, resulting in myocardial maladaptation to pressure overload (29). On the other hand, Danielle et al. perfused rat hearts with the CB2R agonist CB13 in a Langendorff setup and found that CB13 inhibited atrial remodeling caused by tachyarrhythmias (30). In addition, Meeran et al. confirmed that the activation of CB2R had antioxidant, anti-inflammatory and anti-apoptotic effects in rats with myocardial infarction induced by isoproterenol and chronic cardiotoxicity induced by doxorubicin (31, 32). In the current study, considering that Ang II-induced myocardial injury is mainly caused by excessive oxidative stress, we sought to elucidate CB2R-related antioxidant stress pathways. In a series of studies on renal dysfunction, CB2R was found to modulate the expression of NOX (33). However, CB2R has not been studied in the context of myocardial protection, let alone in AF. As expected, after AM1241 treatment, CB2R expression was increased, NOX expression and downstream ox-CaMKII expression were decreased, mitochondrial damage was alleviated, the content of ROS was decreased, and the degree of fibrosis was decreased. Finally, the myocardial conduction velocity increased, and the susceptibility to AF was reduced.

## Conclusion

**FIGURE 6:**
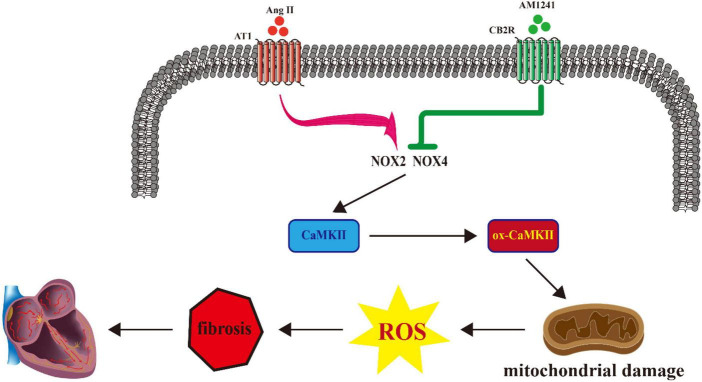
Proposed model describing the role of cannabinoid receptor 2 (CB2R) activation in the treatment of atrial fibrillation (AF).

In summary, we found that in Ang II-infused mice, susceptibility to AF was reduced by pharmacological activation of CB2R, and the signaling pathway involved NOX/CaMKII (Figure 6). Since the onset and persistence of AF are inseparable from oxidative stress, we believe that CB2R will become a potential therapeutic target for the treatment of AF.

Nevertheless, our research had some limitations. More accurate knockout, overexpression, and other interventions for in-depth research on the molecular mechanism are needed. Although the Ang II-induced mouse model of AF has been widely used and is representative (34), the role of the endocannabinoid system in AF should be validated in more animal models, such as transverse aortic constriction (TAC) mice. In addition, AM1241 is not a myocardial-specific CB2R agonist, and it is difficult to rule out the possibility that the drug affected the heart through its effects on other systems. Research on myocardial-specific CB2R agonists is urgently needed.

Although the current study was performed only on an Ang II-induced mouse model, the positive results are very promising, and the potential psychiatric side effects of CB1R were avoided. We believe CB2R agonists will be beneficial for the treatment of AF.

## Data availability statement

The raw data supporting the conclusions of this article will be made available by the authors, without undue reservation.

## Ethics statement

The animal study was reviewed and approved by the Animal Care Committee of General Hospital of Northern Theater Command.

## Author contributions

HW and LY designed the experiment and checked the manuscript. DX and CX performed the experiment, acquired the data, and drafted the manuscript. XX, YX, JZ, and TH analyzed and interpreted the data. ZW, QZ, ZZ, and YH revised the manuscript and supervised the study. All authors read and approved the final manuscript.
