# Supplementary figures and images for: Activation of cannabinoid receptor 2 attenuates Angiotensin II-induced atrial fibrillation via a potential NOX/CaMKII mechanism
Source: Front Cardiovasc Med. 2022 Oct 14;9:968014. doi: 10.3389/fcvm.2022.968014 (PMC9616165; doi:10.3389/fcvm.2022.968014)

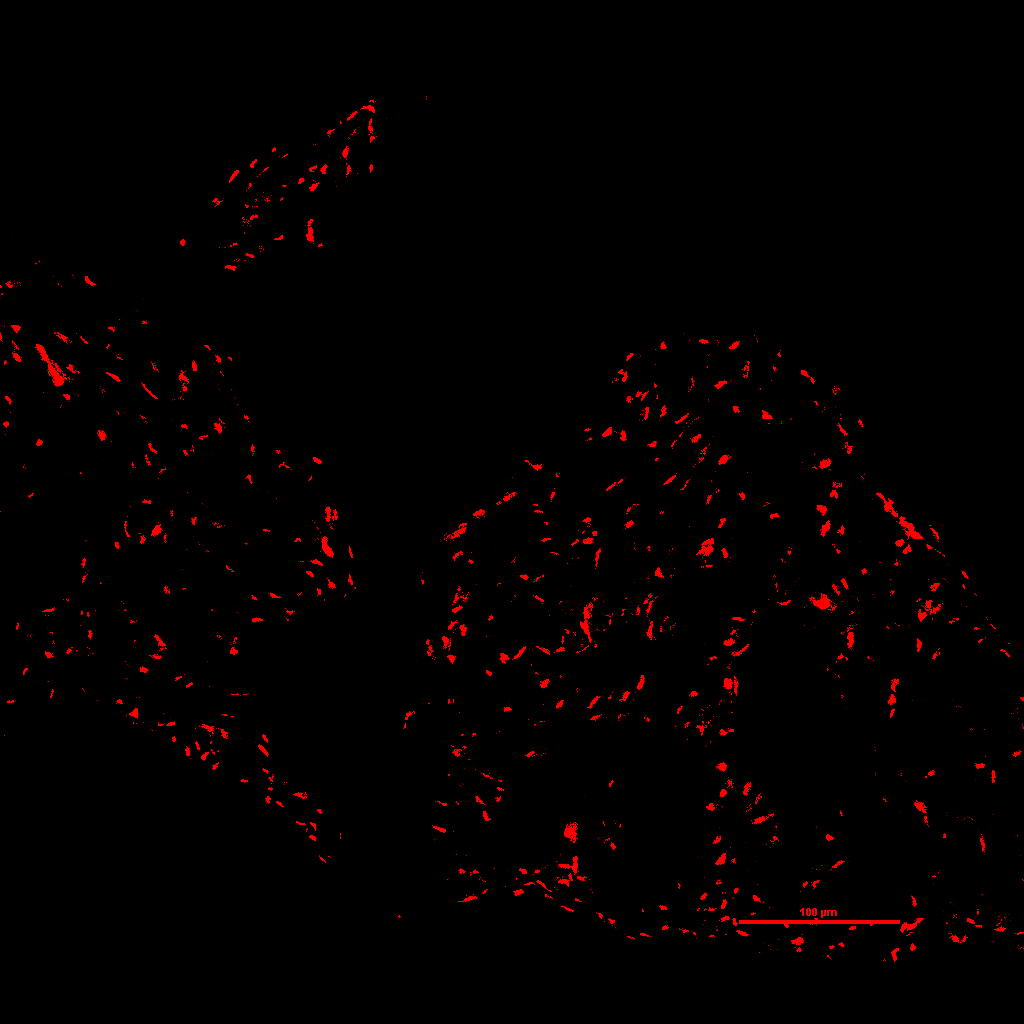

Supplement: Supplementary file 1 [file Data_Sheet_1.ZIP › DHE/Ang II.tif]

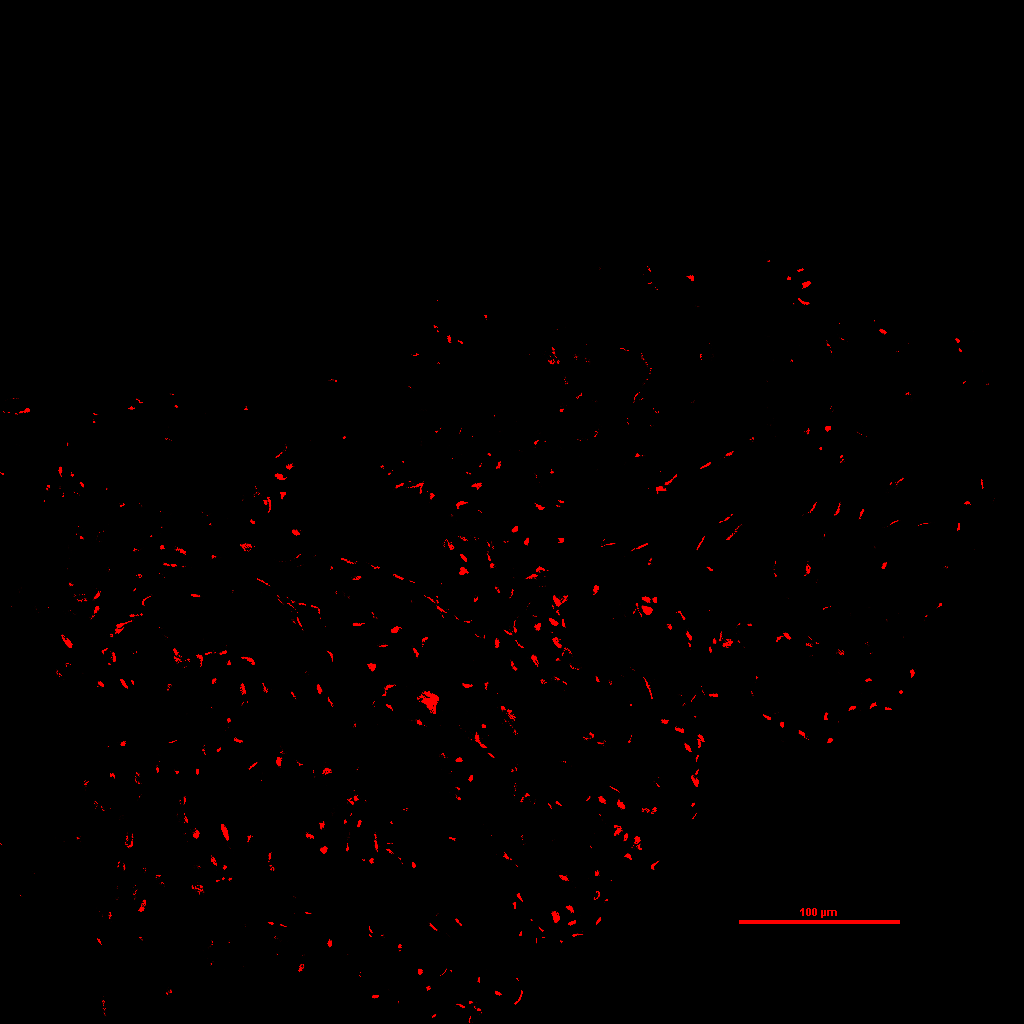

Supplement: Supplementary file 1 [file Data_Sheet_1.ZIP › DHE/Ang II+AM1241.tif]

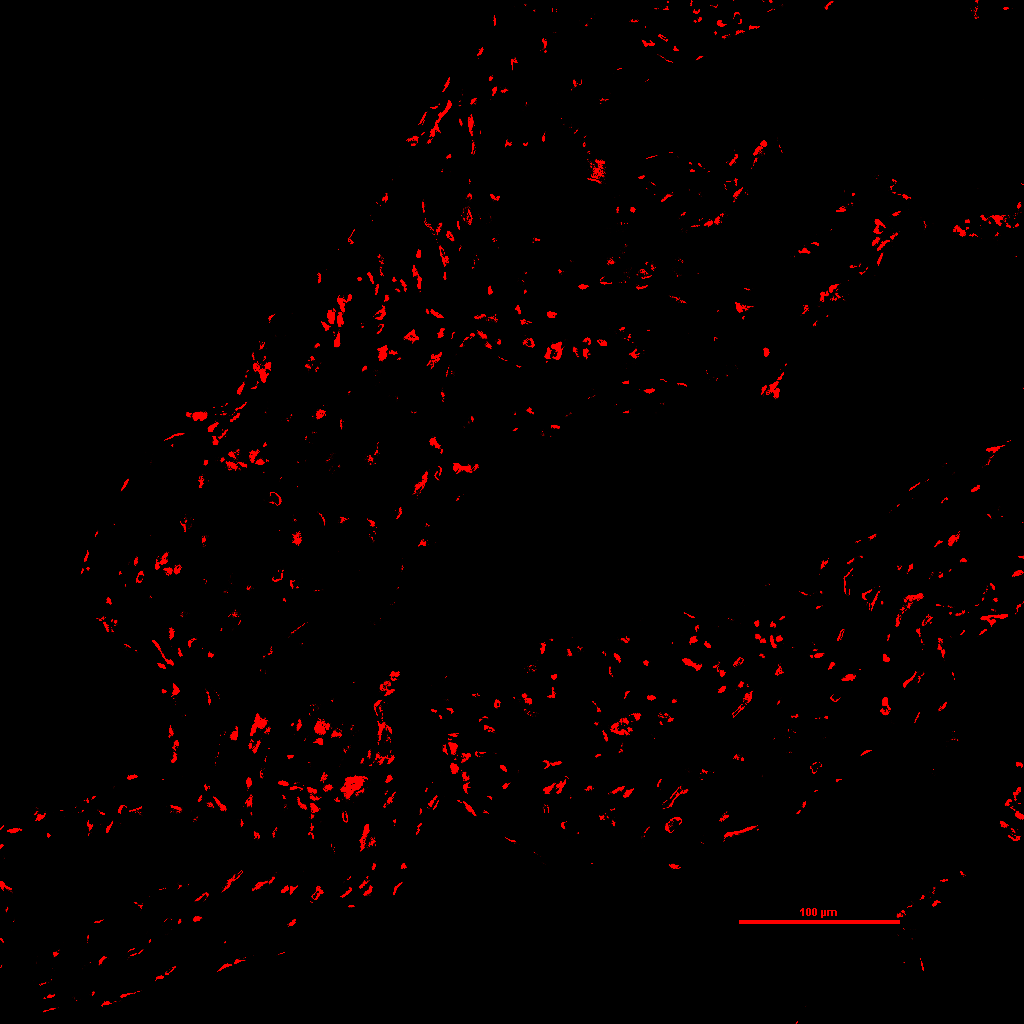

Supplement: Supplementary file 1 [file Data_Sheet_1.ZIP › DHE/Ang II+AM630.tif]

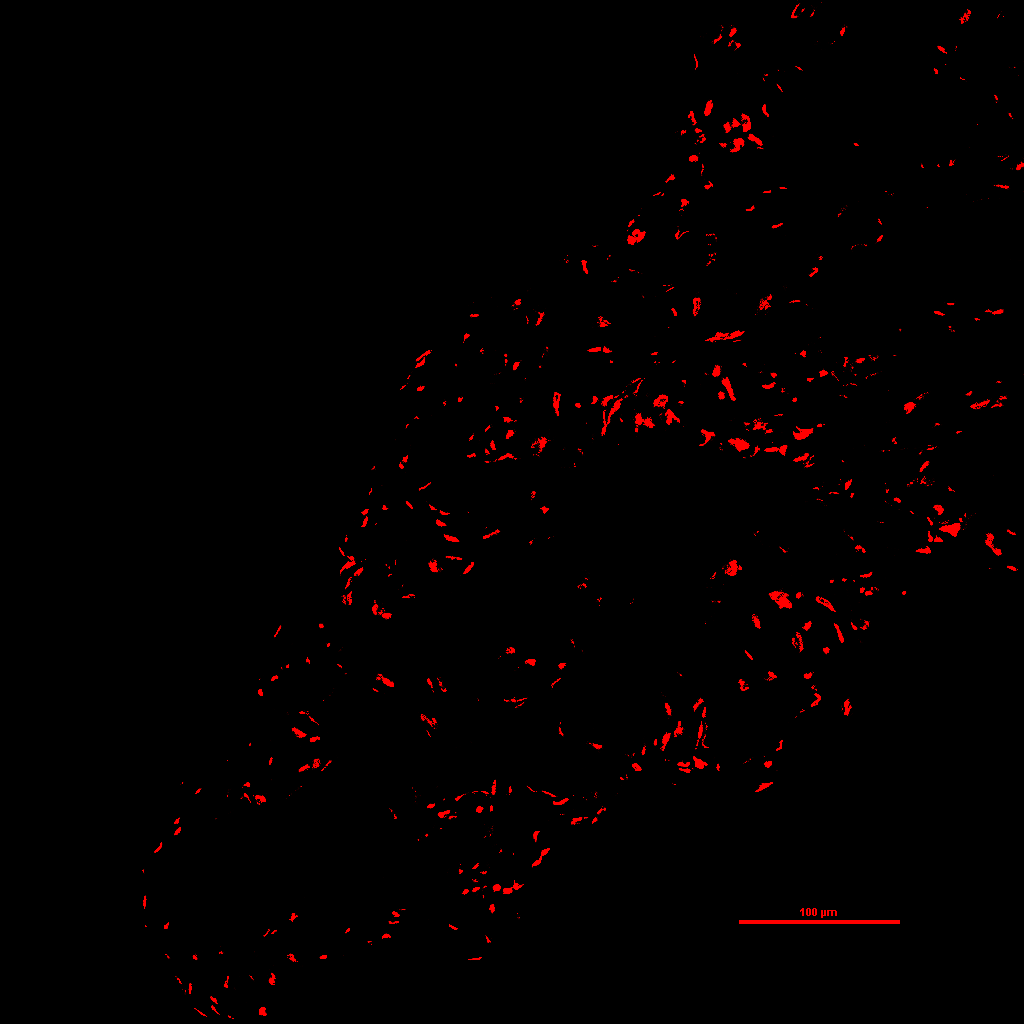

Supplement: Supplementary file 1 [file Data_Sheet_1.ZIP › DHE/Ang II+Veh.tif]

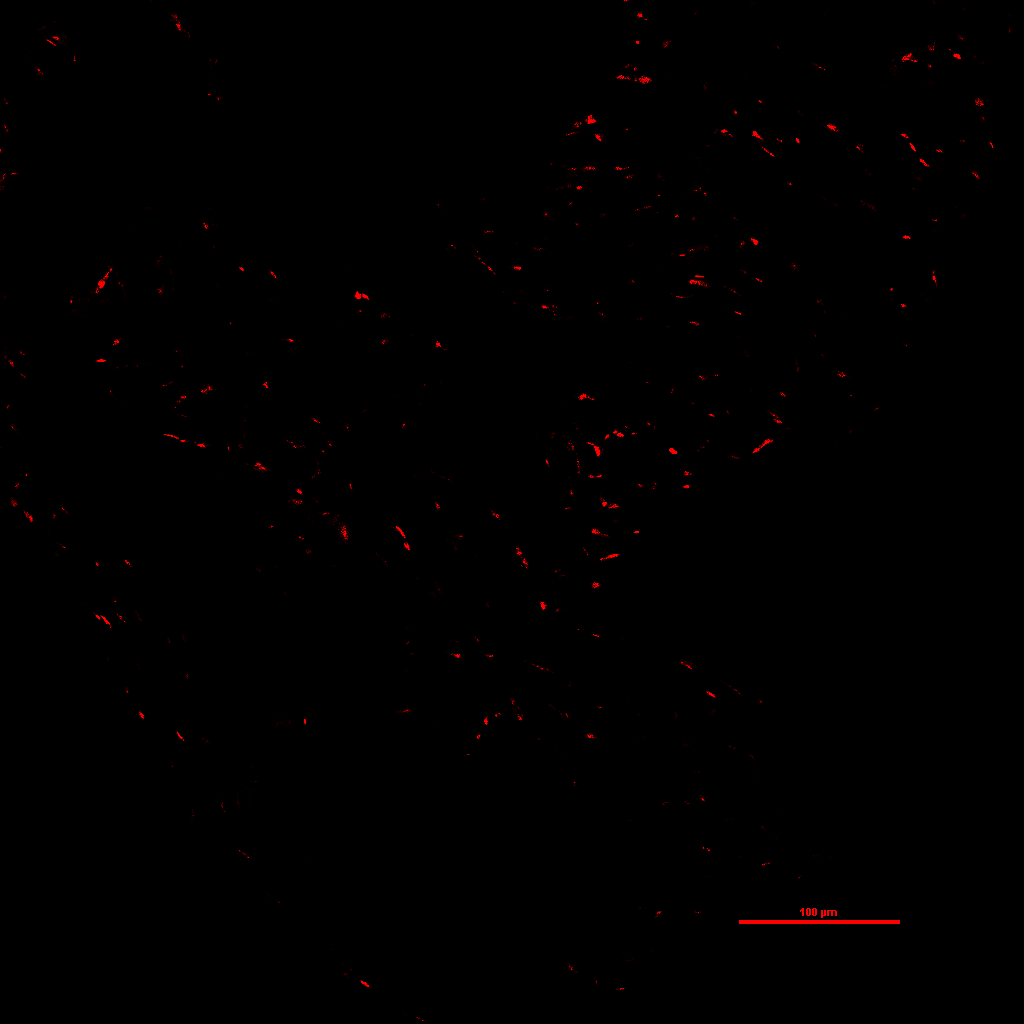

Supplement: Supplementary file 1 [file Data_Sheet_1.ZIP › DHE/Control.tif]

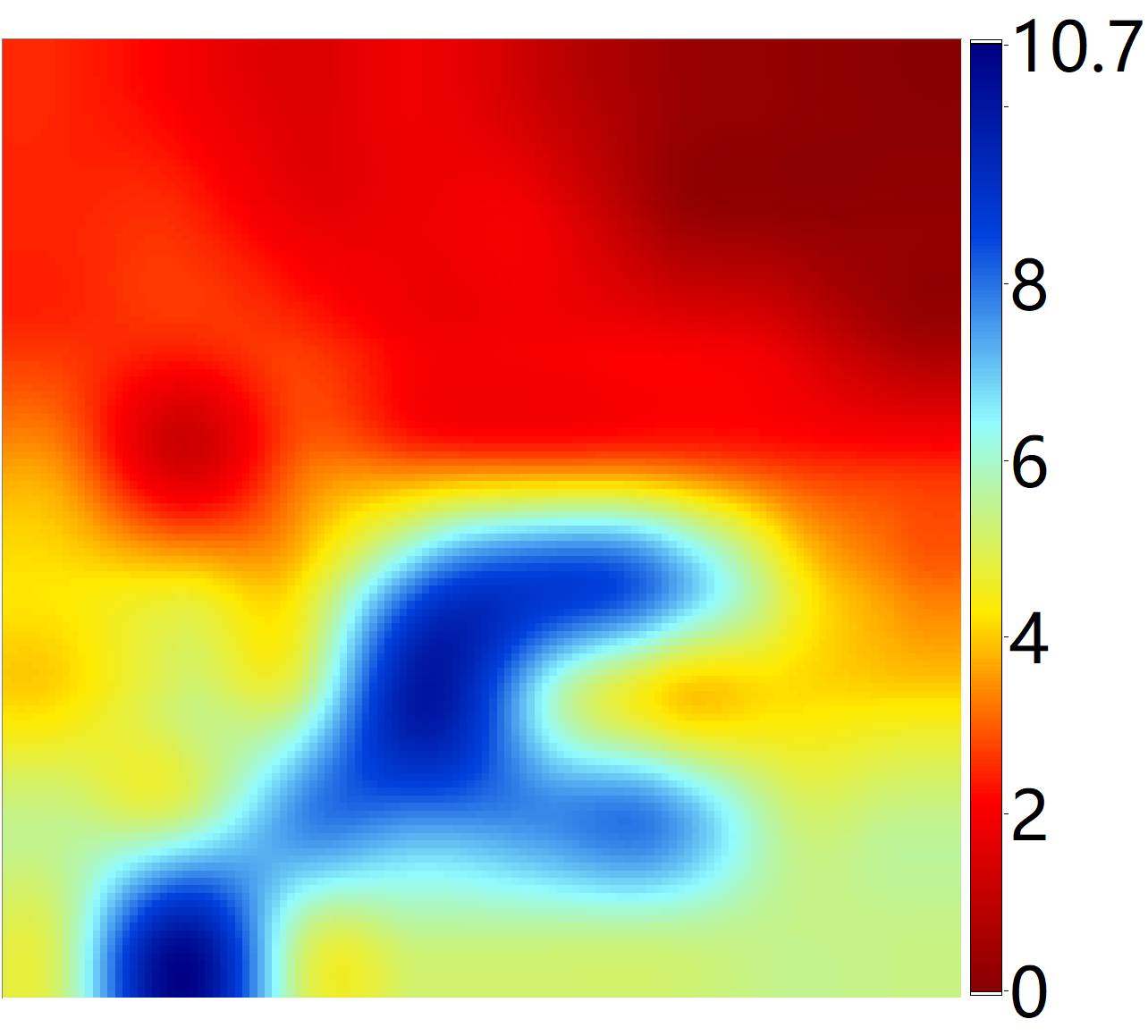

Supplement: Supplementary file 3 [file Data_Sheet_3.ZIP › Mapping/Ang II.tif]

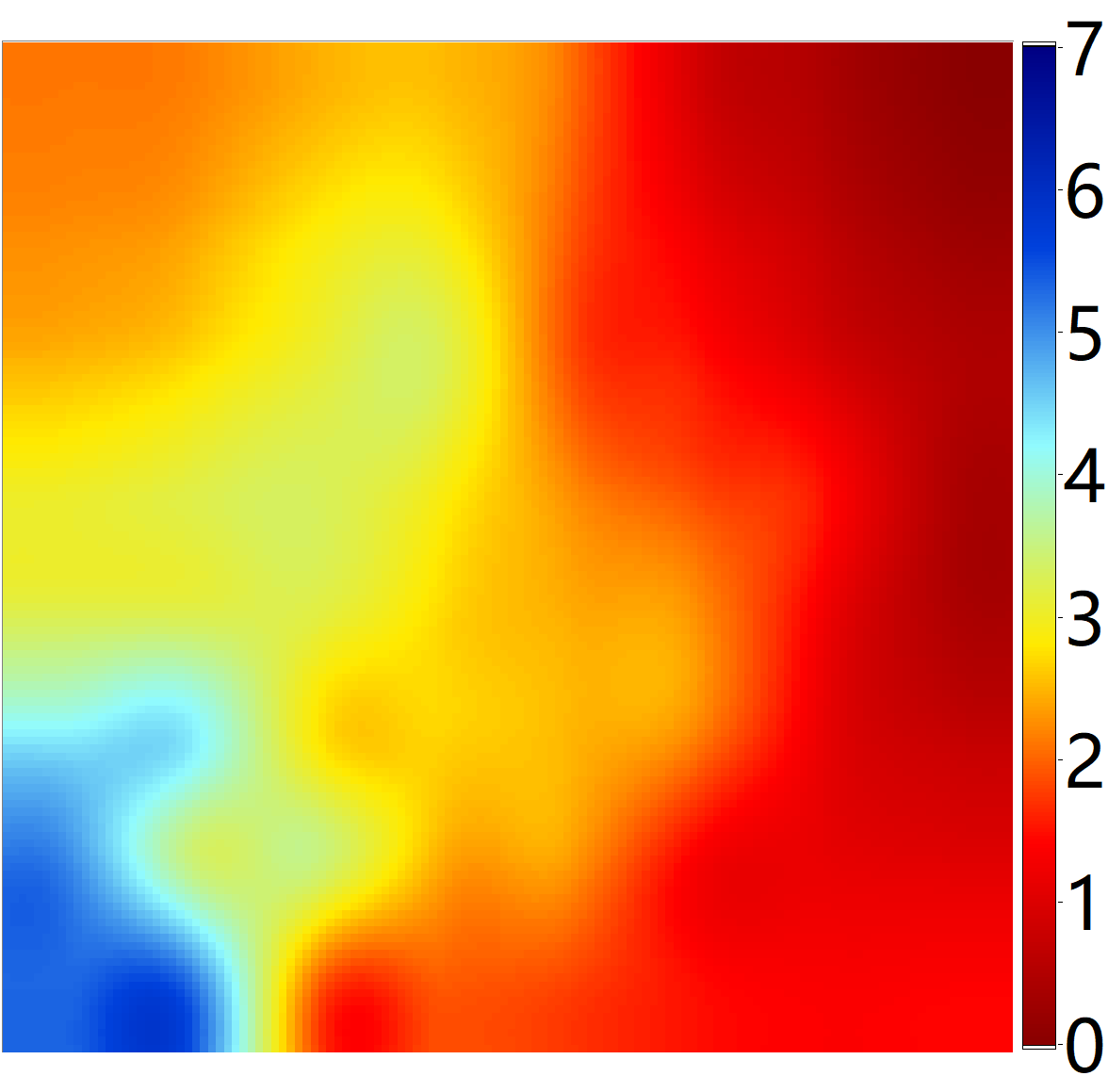

Supplement: Supplementary file 3 [file Data_Sheet_3.ZIP › Mapping/Ang II+AM1241.tif]

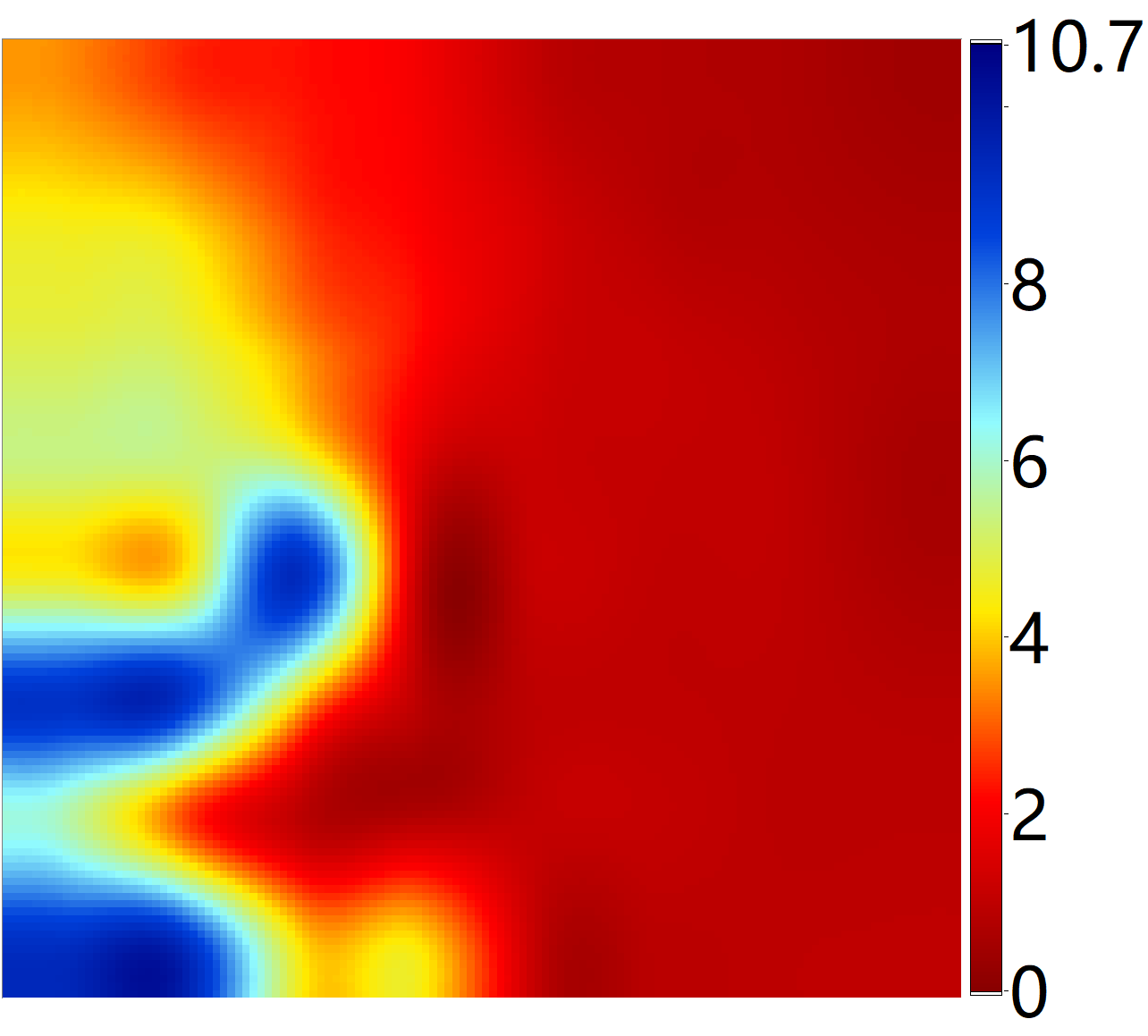

Supplement: Supplementary file 3 [file Data_Sheet_3.ZIP › Mapping/Ang II+AM630.tif]

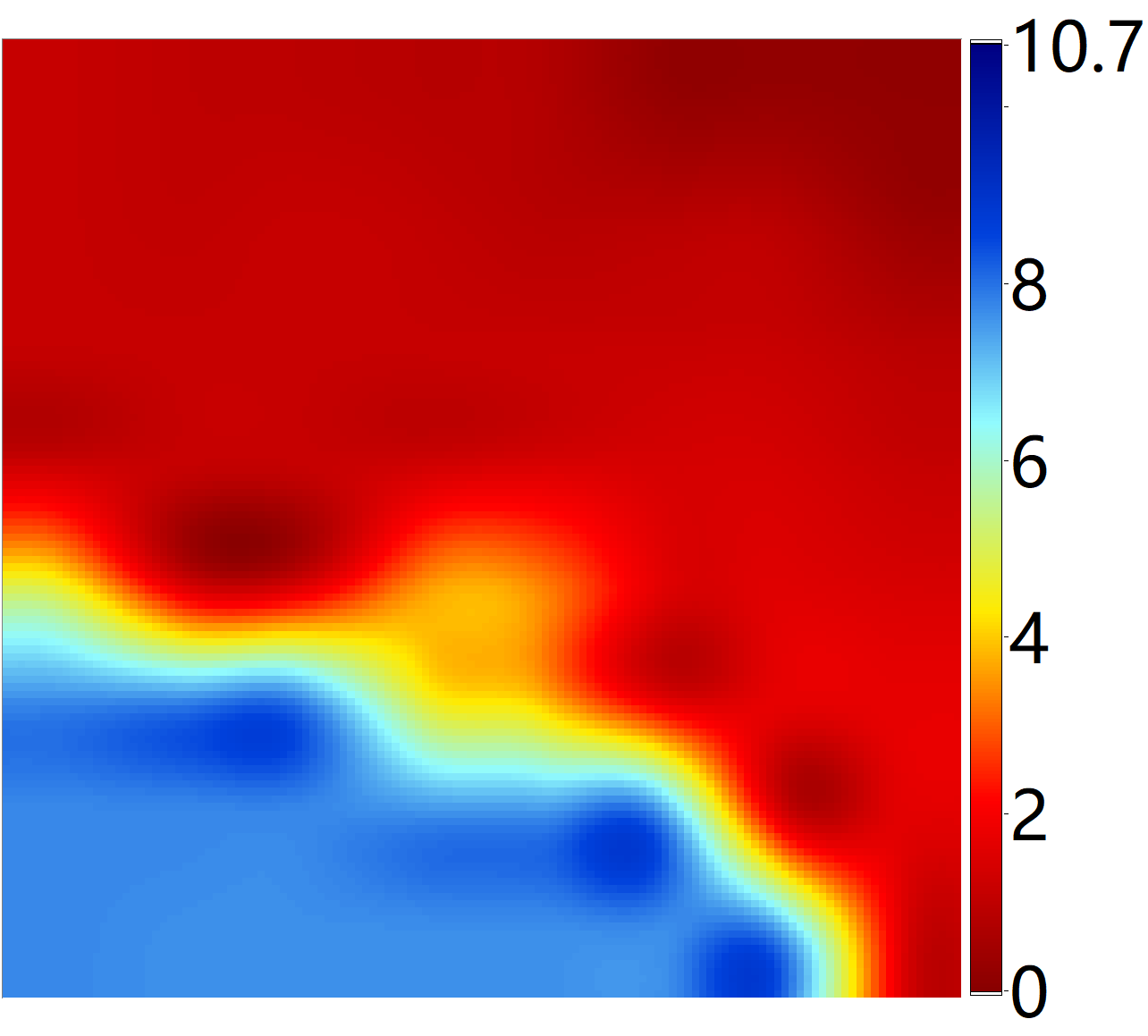

Supplement: Supplementary file 3 [file Data_Sheet_3.ZIP › Mapping/Ang II+Veh.tif]

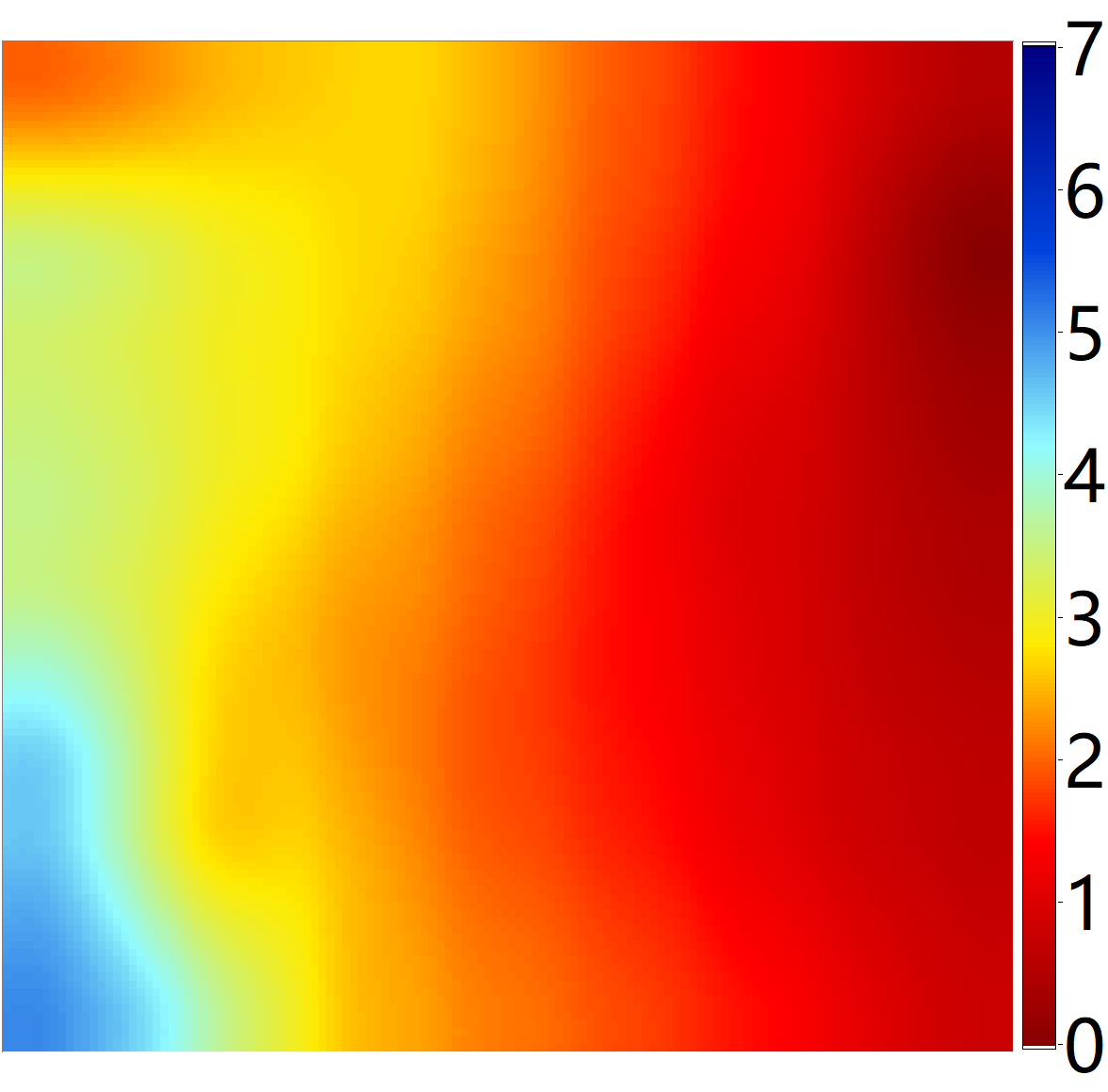

Supplement: Supplementary file 3 [file Data_Sheet_3.ZIP › Mapping/Control.tif]

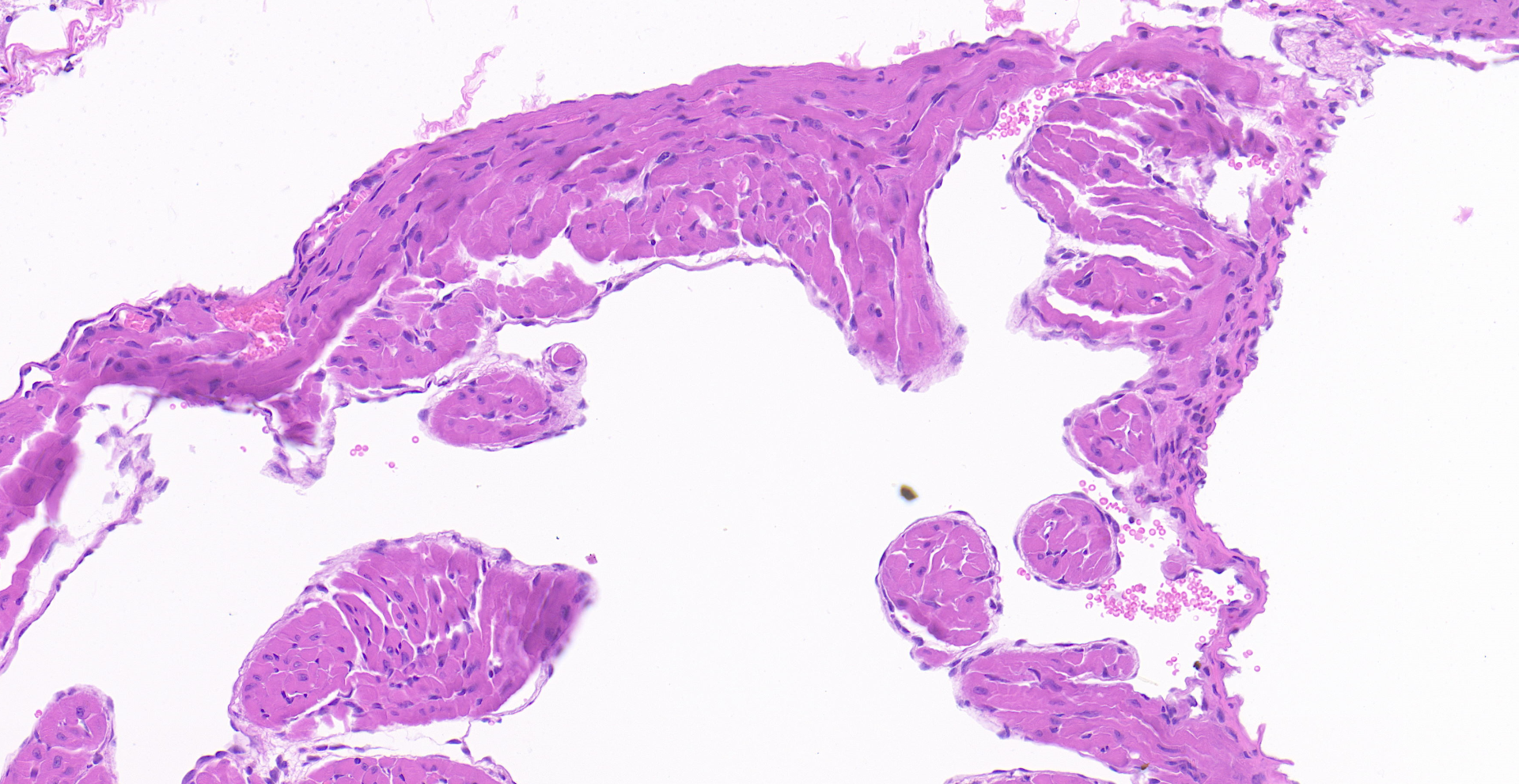

Supplement: Supplementary file 4 [file Data_Sheet_4.ZIP › HE/Ang II.tif]

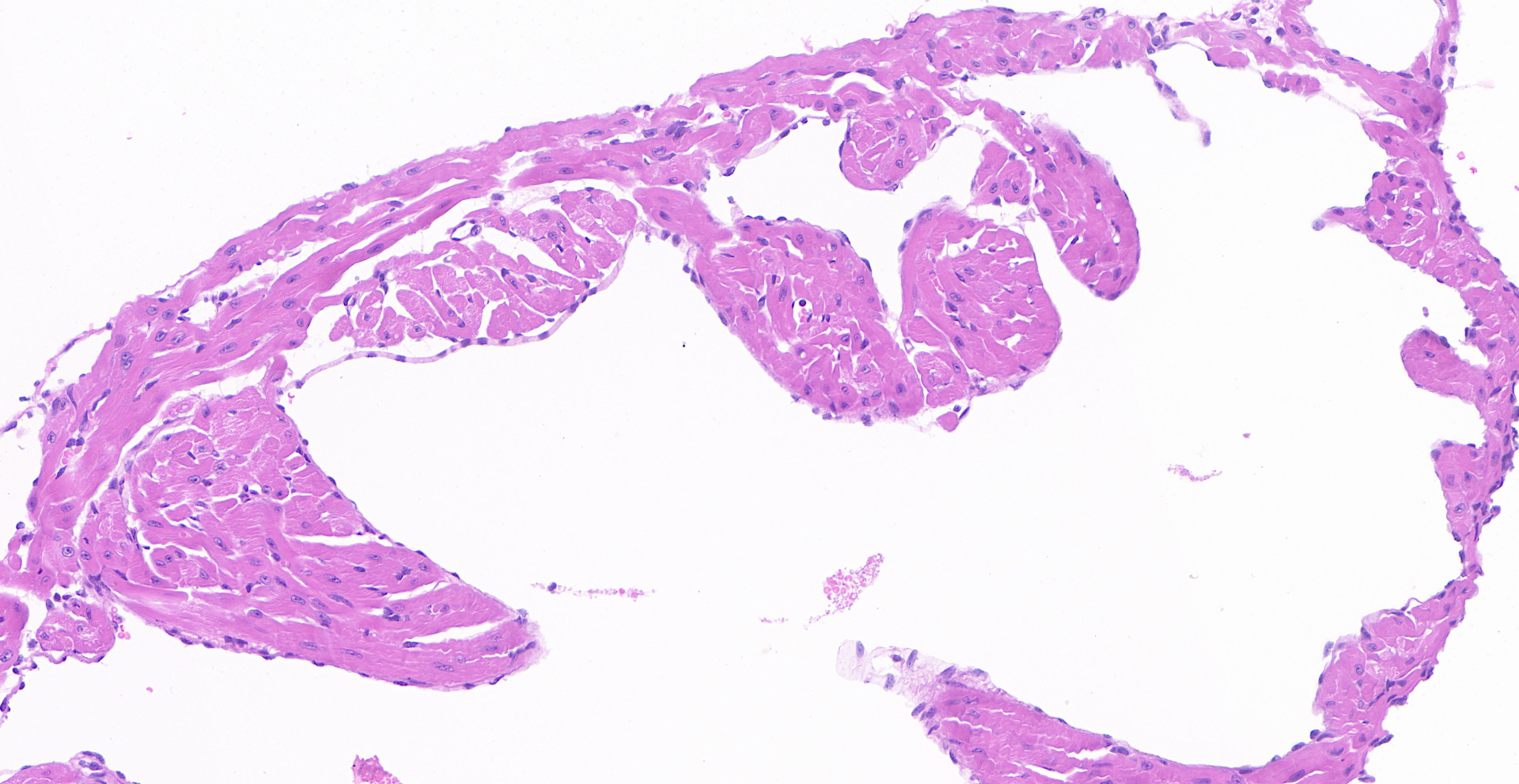

Supplement: Supplementary file 4 [file Data_Sheet_4.ZIP › HE/Ang II+AM1241.tif]

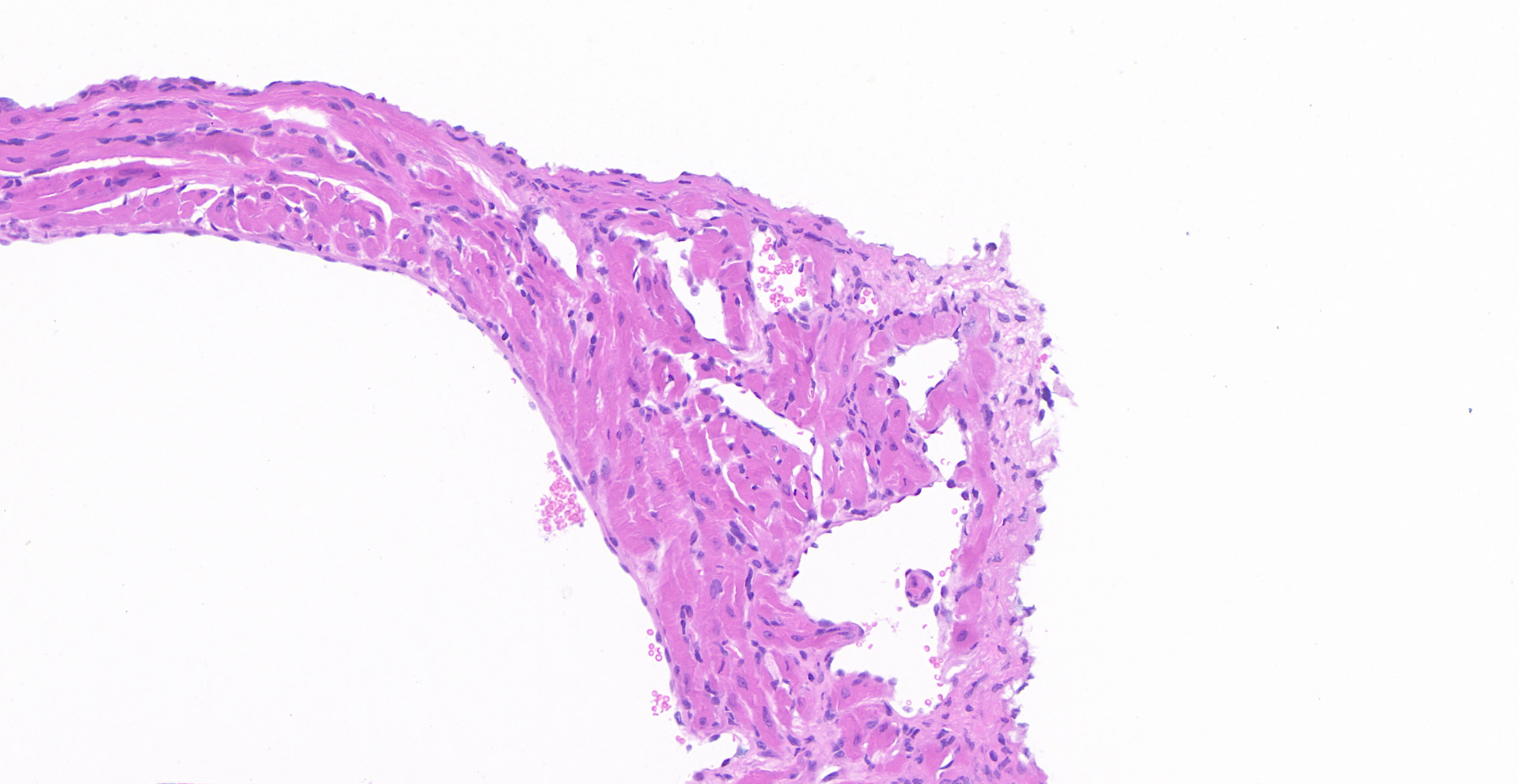

Supplement: Supplementary file 4 [file Data_Sheet_4.ZIP › HE/Ang II+AM630.tif]

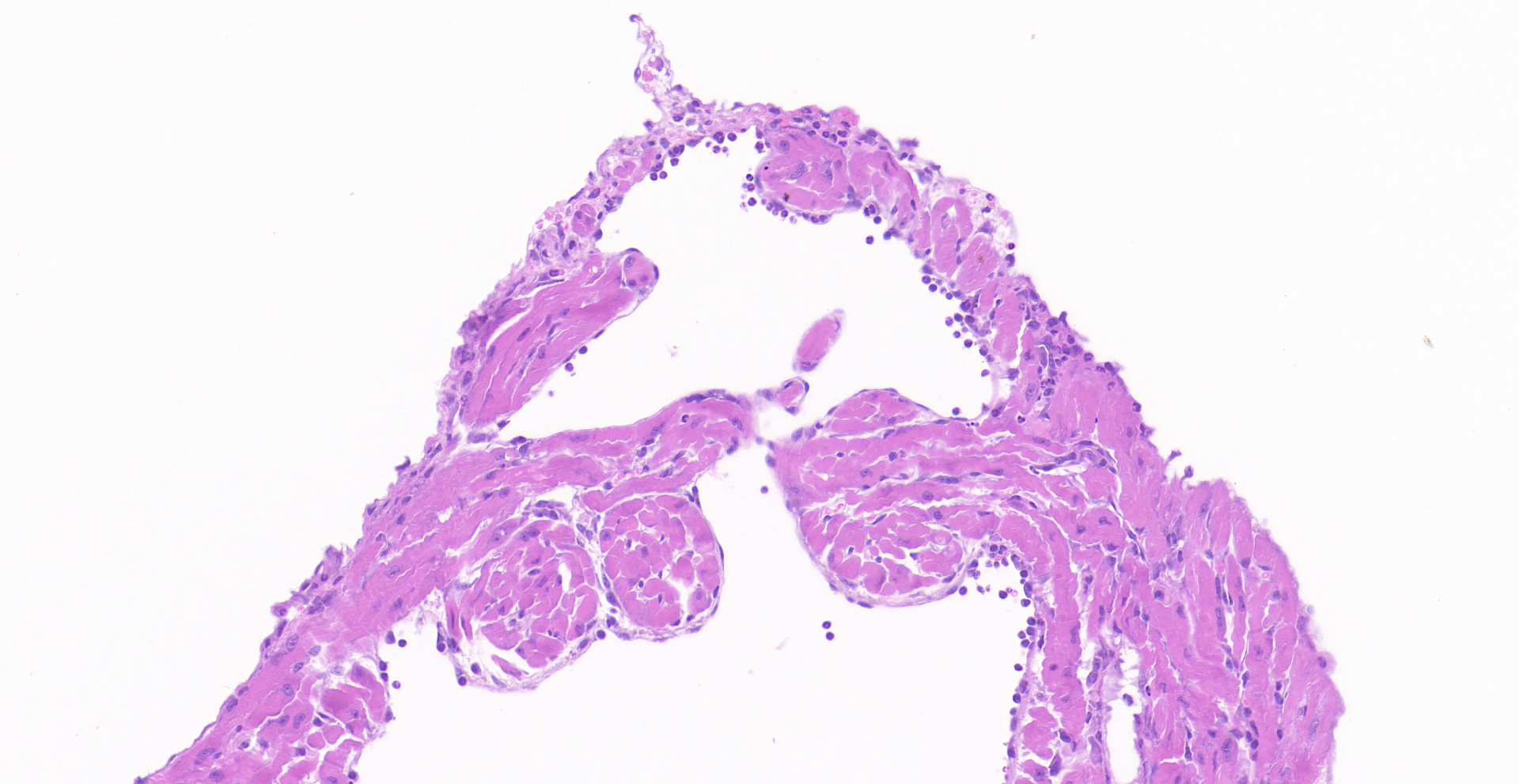

Supplement: Supplementary file 4 [file Data_Sheet_4.ZIP › HE/Ang II+Veh.tif]

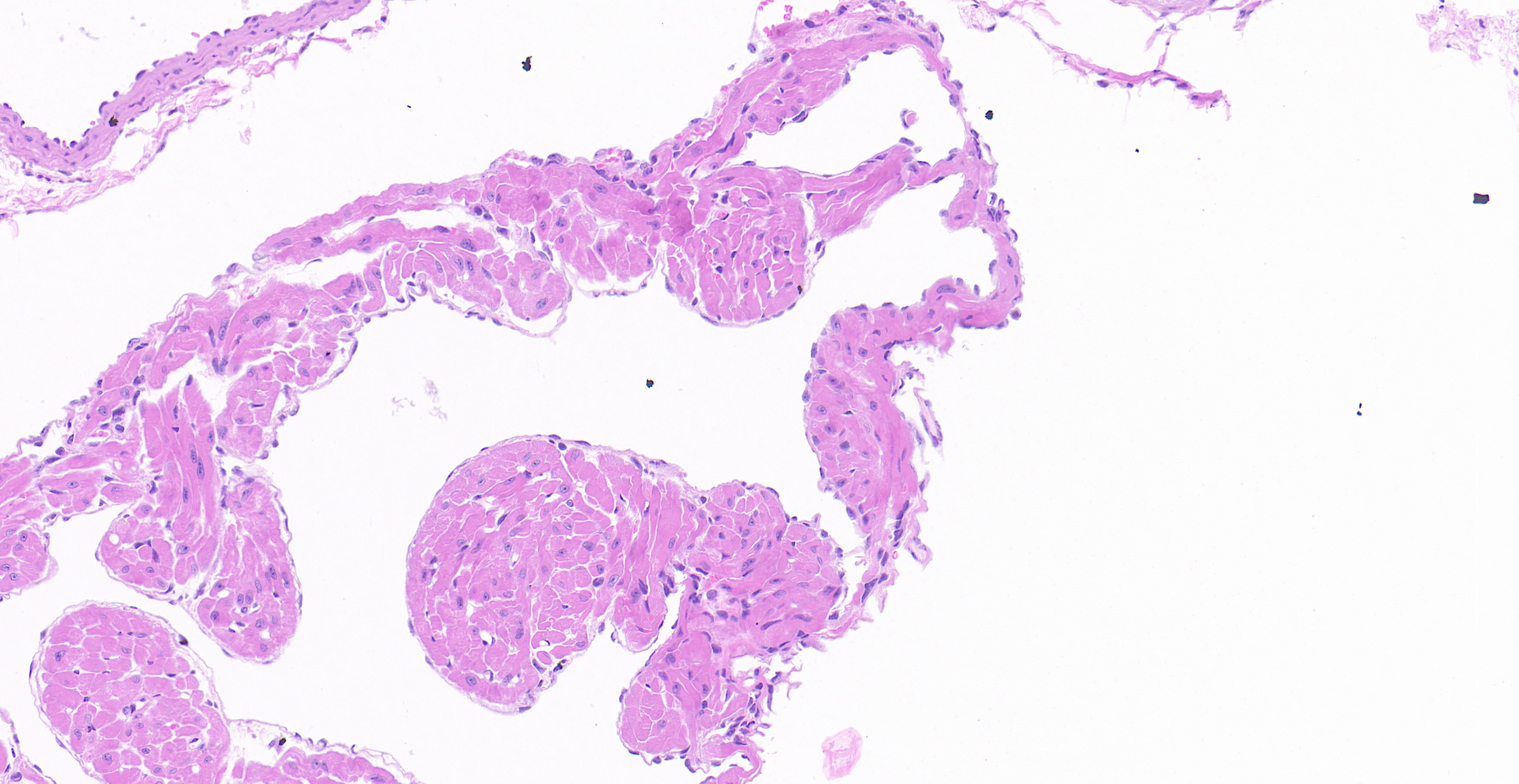

Supplement: Supplementary file 4 [file Data_Sheet_4.ZIP › HE/Control.tif]

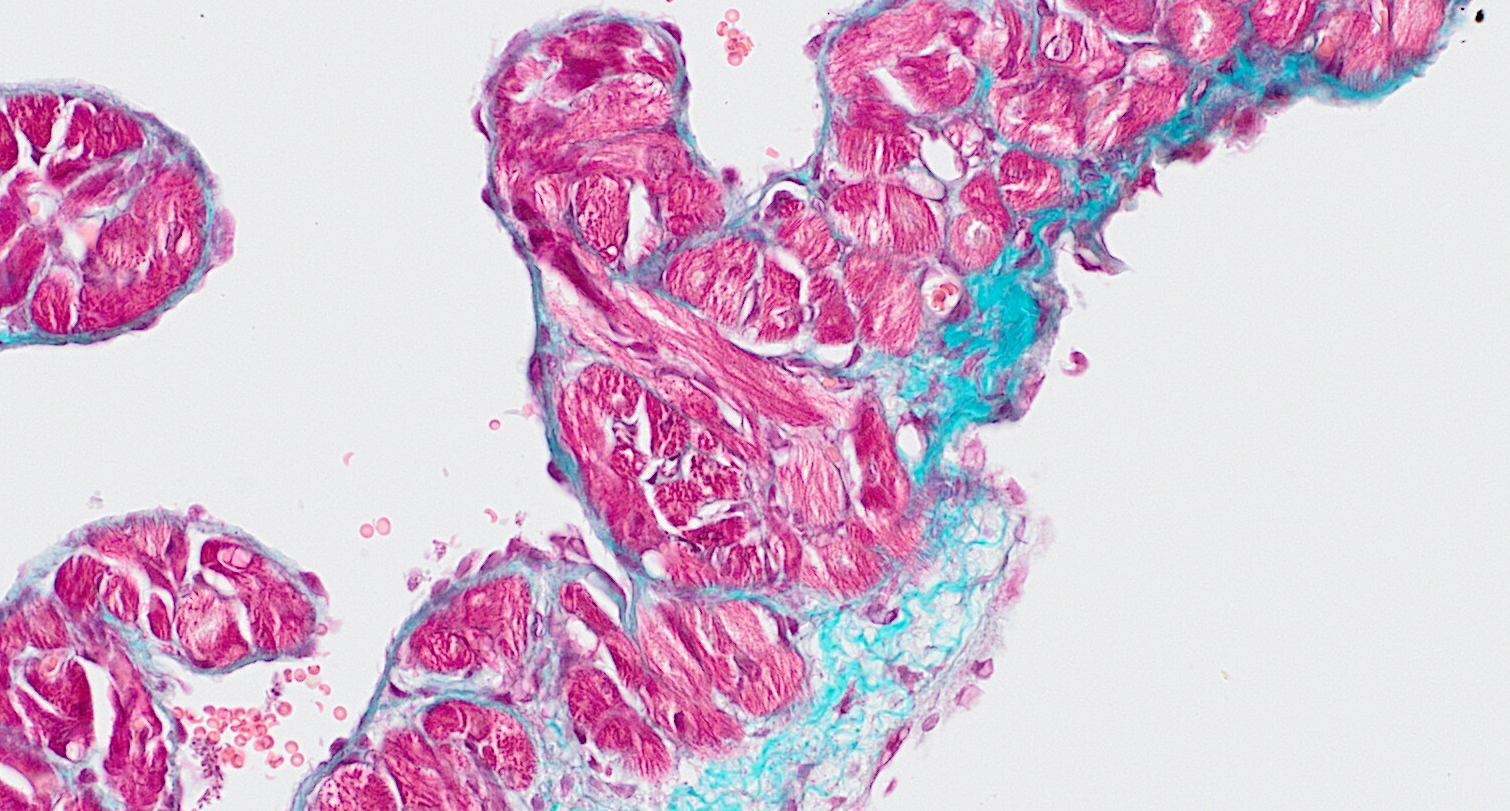

Supplement: Supplementary file 5 [file Data_Sheet_5.ZIP › Masson/Ang II.tif]

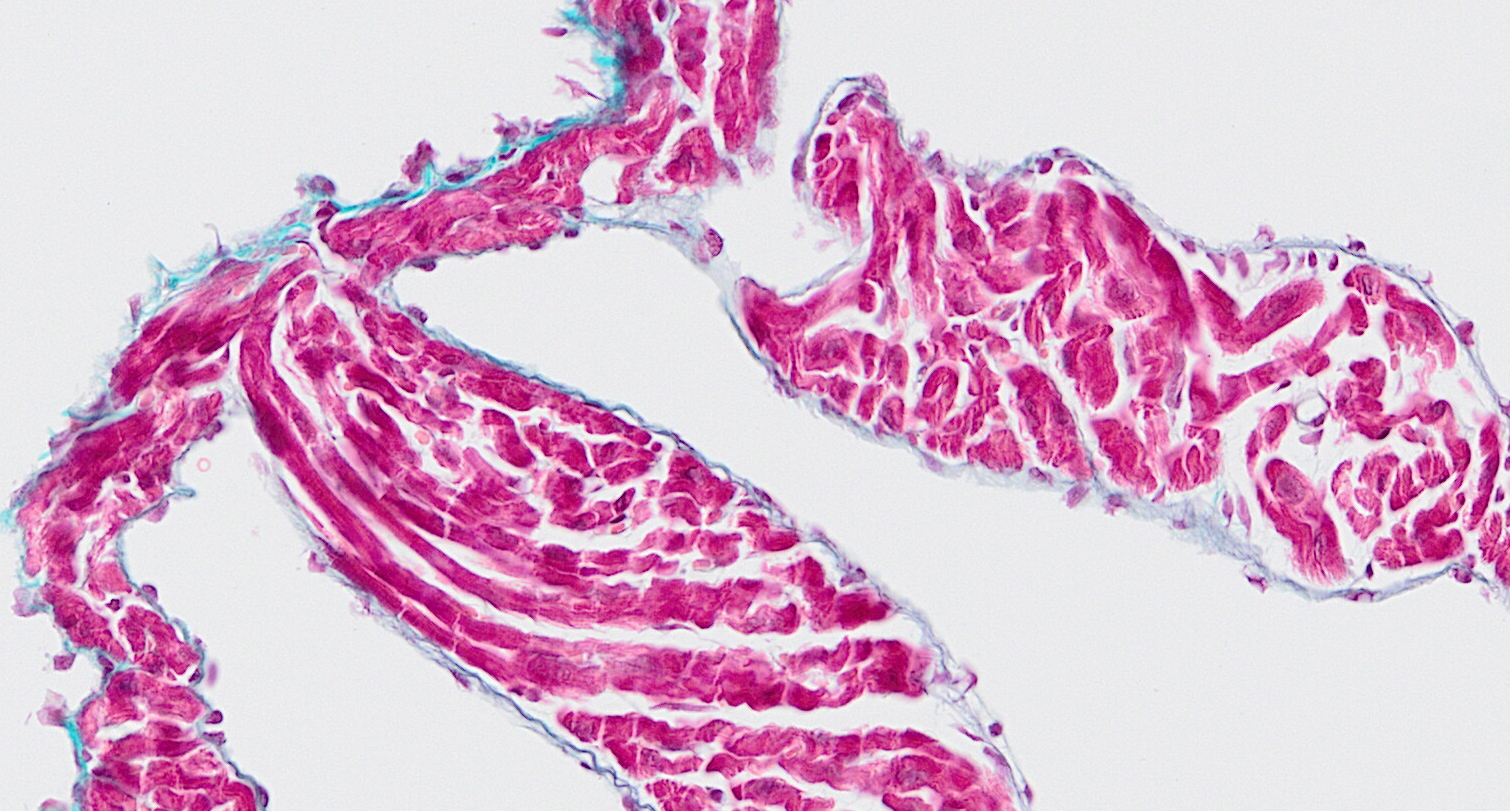

Supplement: Supplementary file 5 [file Data_Sheet_5.ZIP › Masson/Ang II+AM1241.tif]

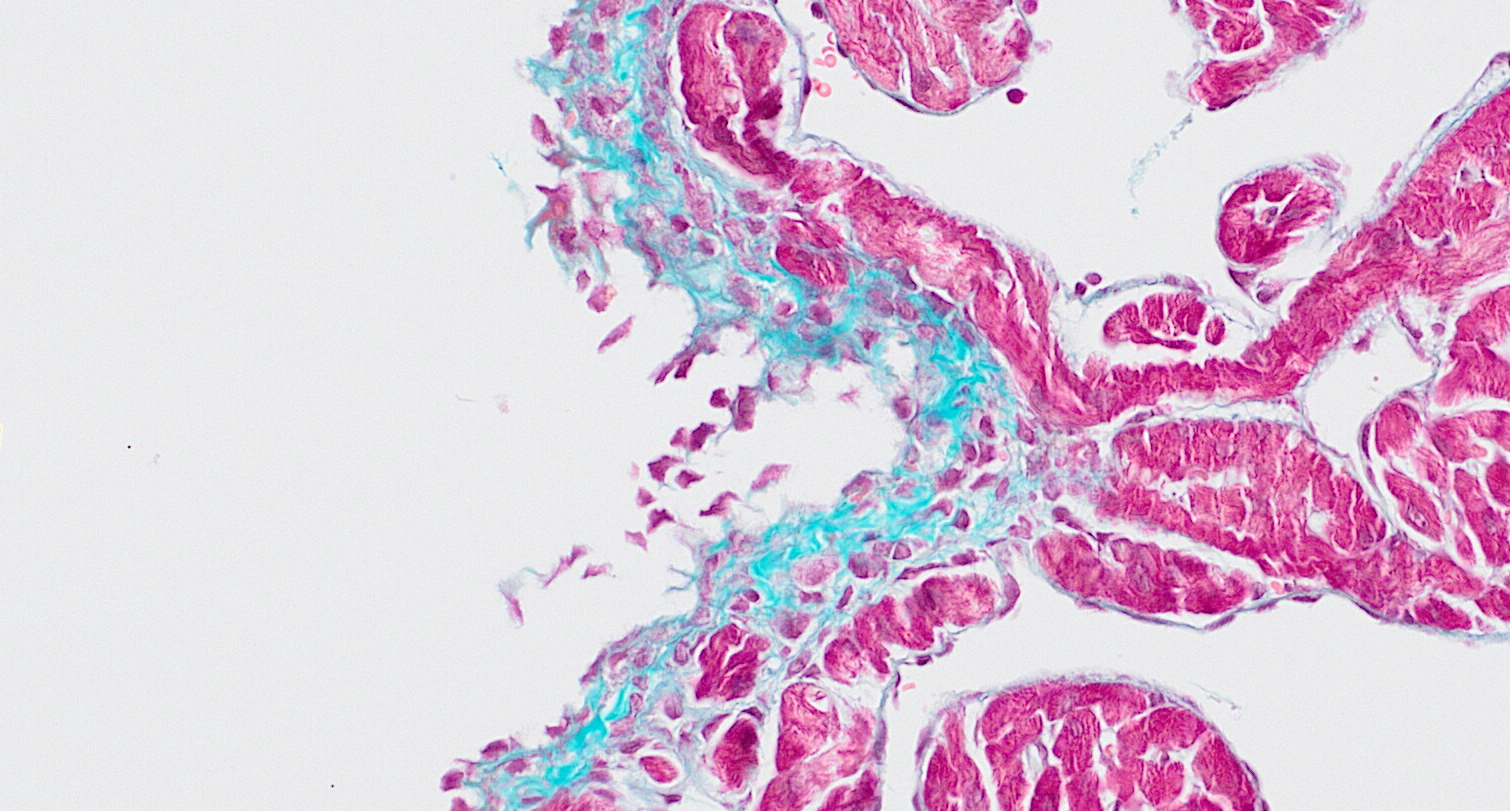

Supplement: Supplementary file 5 [file Data_Sheet_5.ZIP › Masson/Ang II+AM630.tif]

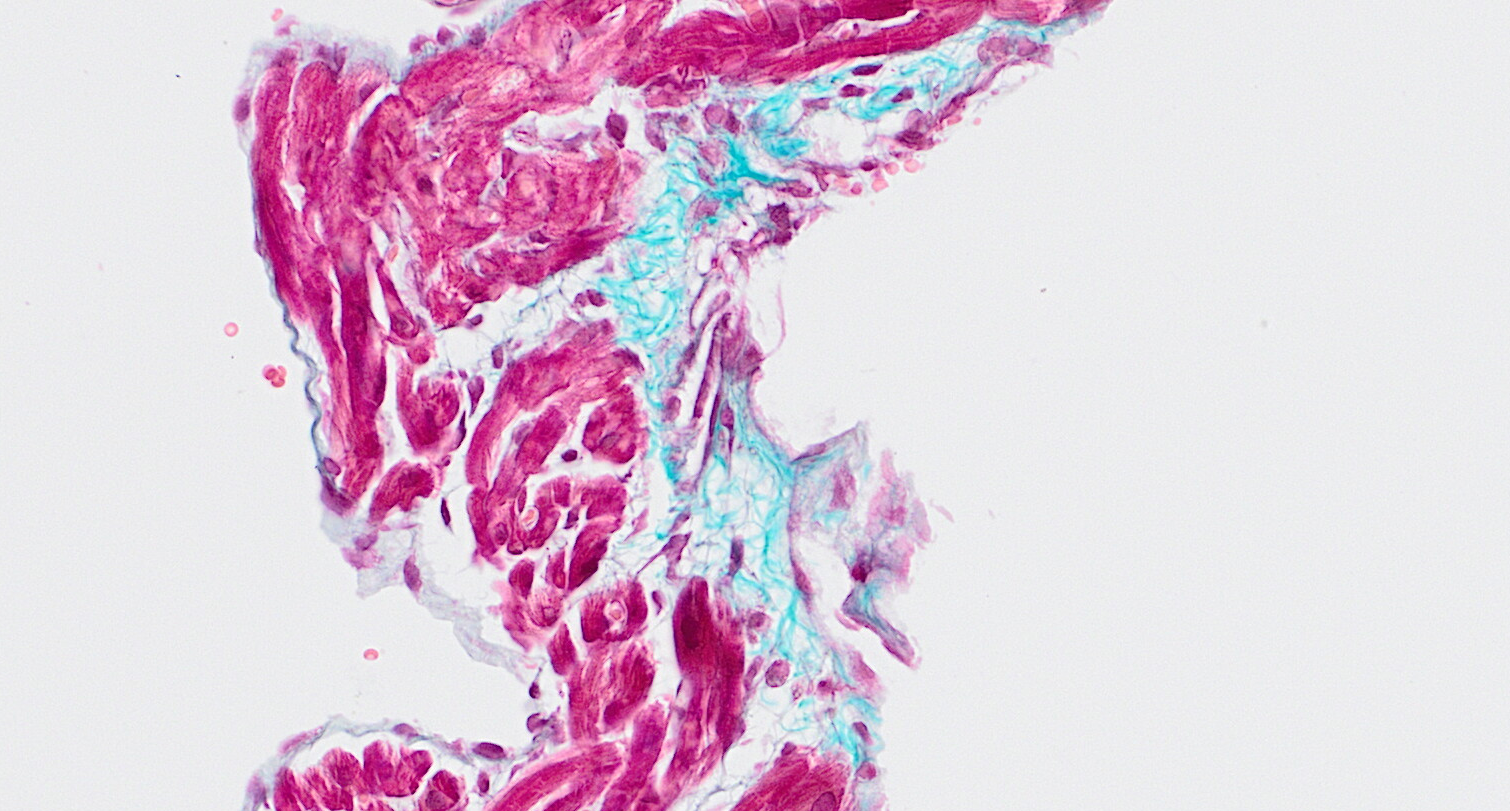

Supplement: Supplementary file 5 [file Data_Sheet_5.ZIP › Masson/Ang II+Veh.tif]

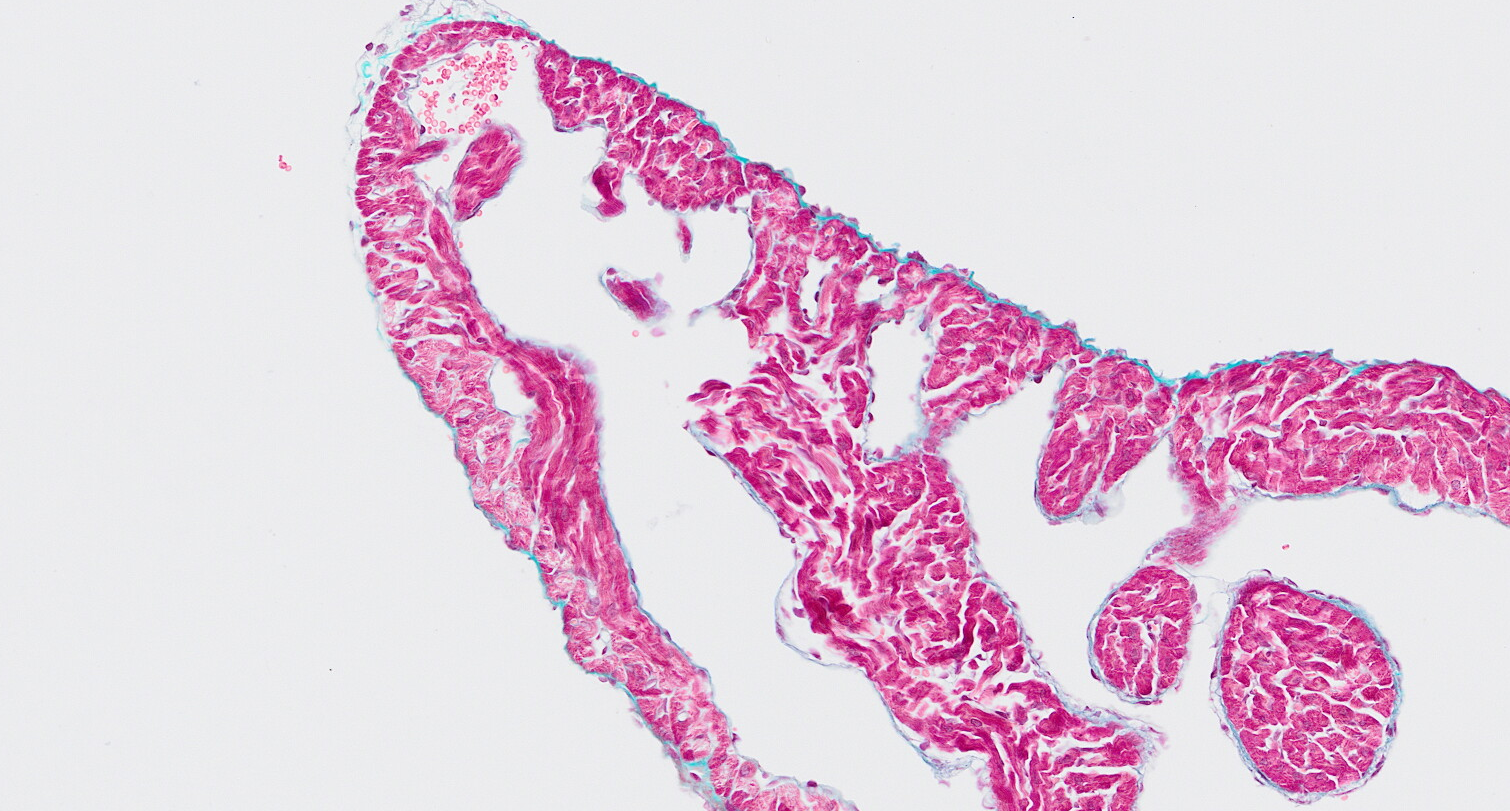

Supplement: Supplementary file 5 [file Data_Sheet_5.ZIP › Masson/Control.tif]

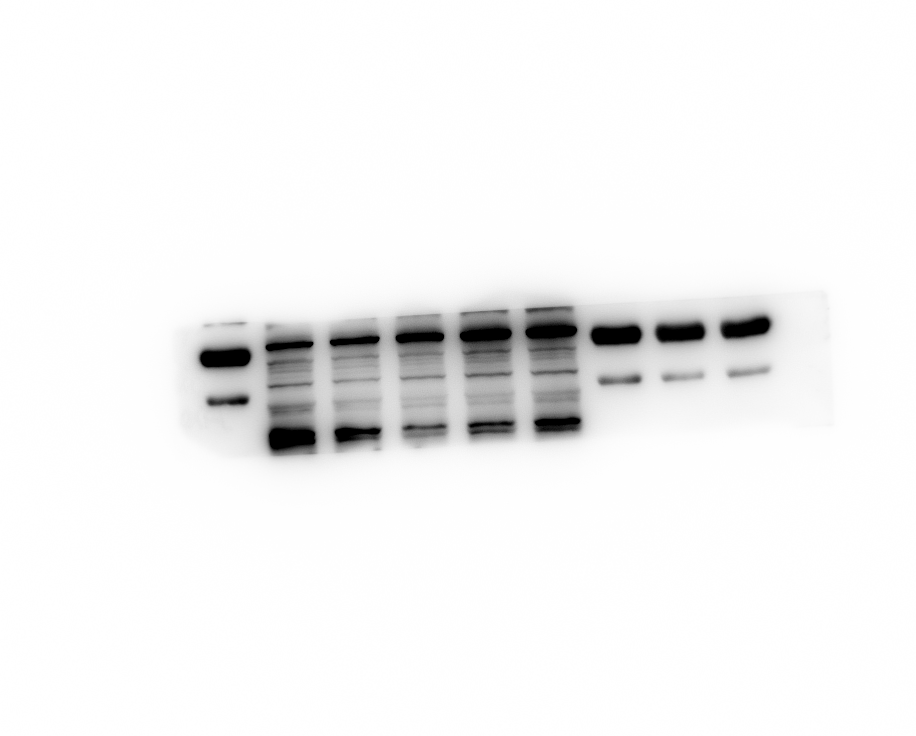

Supplement: Supplementary file 6 [file Data_Sheet_6.ZIP › WB/CaMKII.tif]

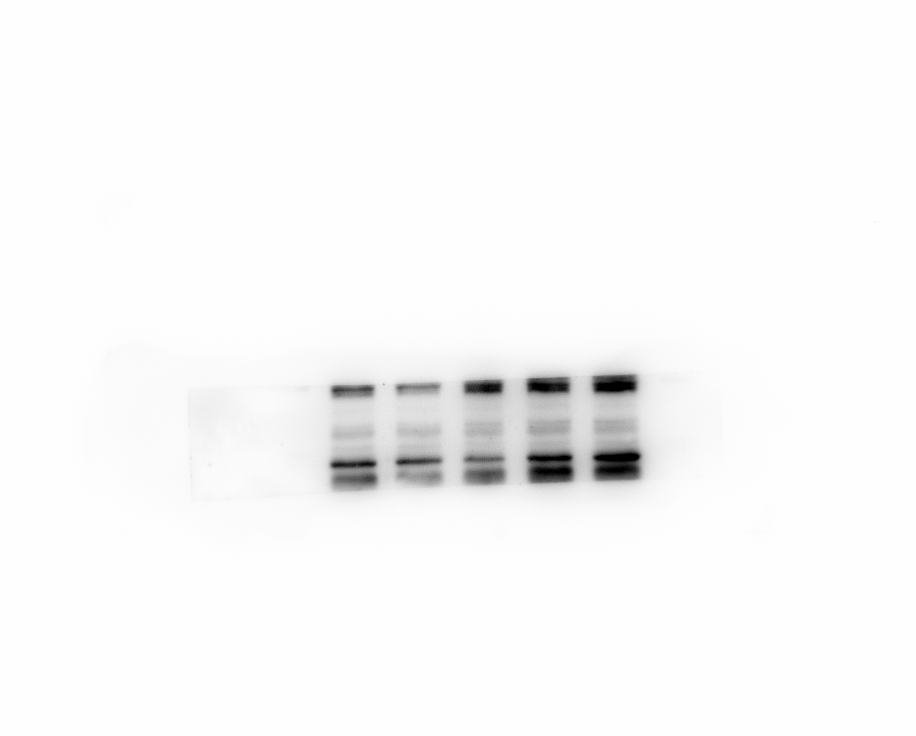

Supplement: Supplementary file 6 [file Data_Sheet_6.ZIP › WB/CB2R.tif]

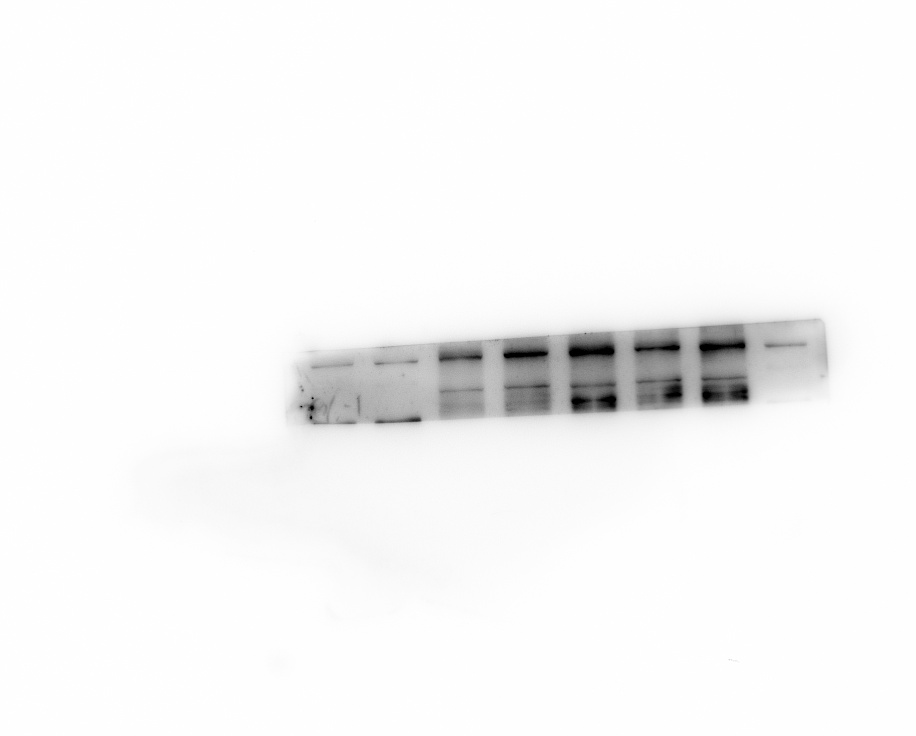

Supplement: Supplementary file 6 [file Data_Sheet_6.ZIP › WB/Collagen I.tif]

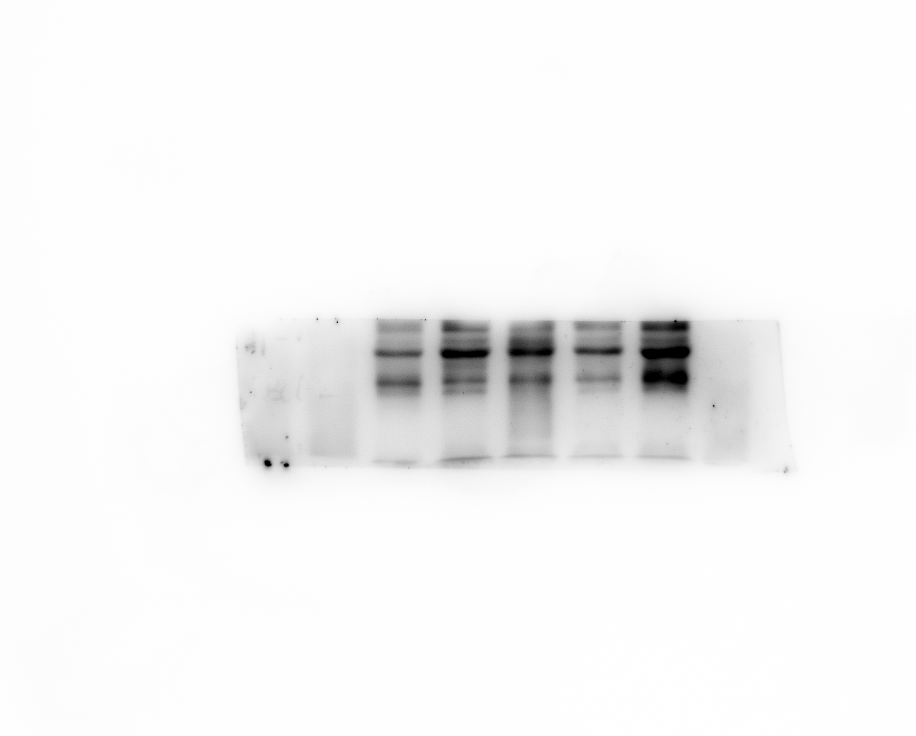

Supplement: Supplementary file 6 [file Data_Sheet_6.ZIP › WB/Collagen III.tif]

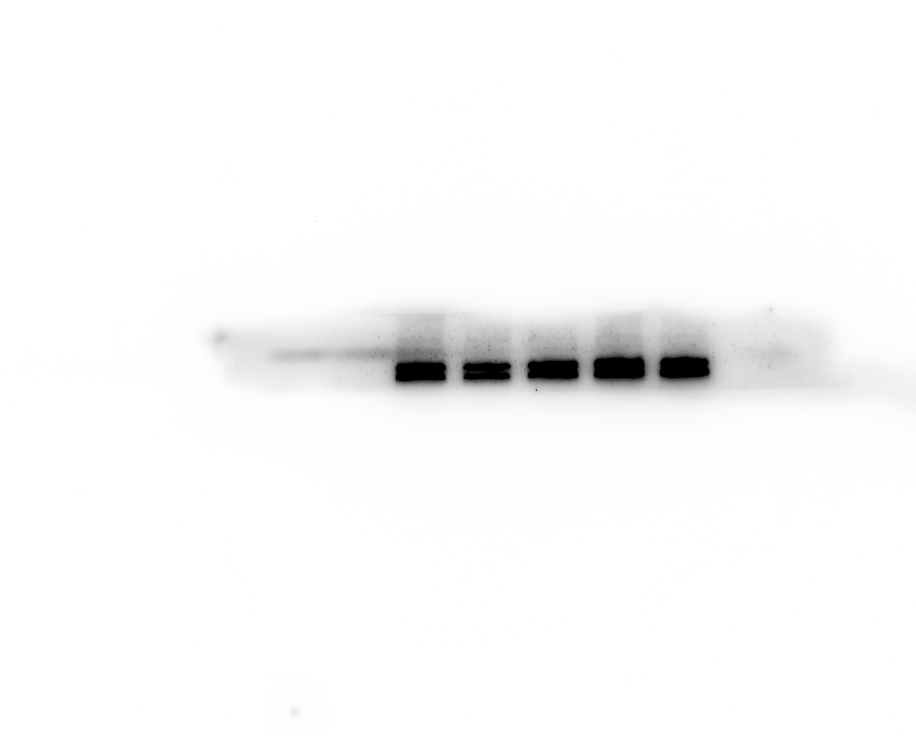

Supplement: Supplementary file 6 [file Data_Sheet_6.ZIP › WB/Drp1.tif]

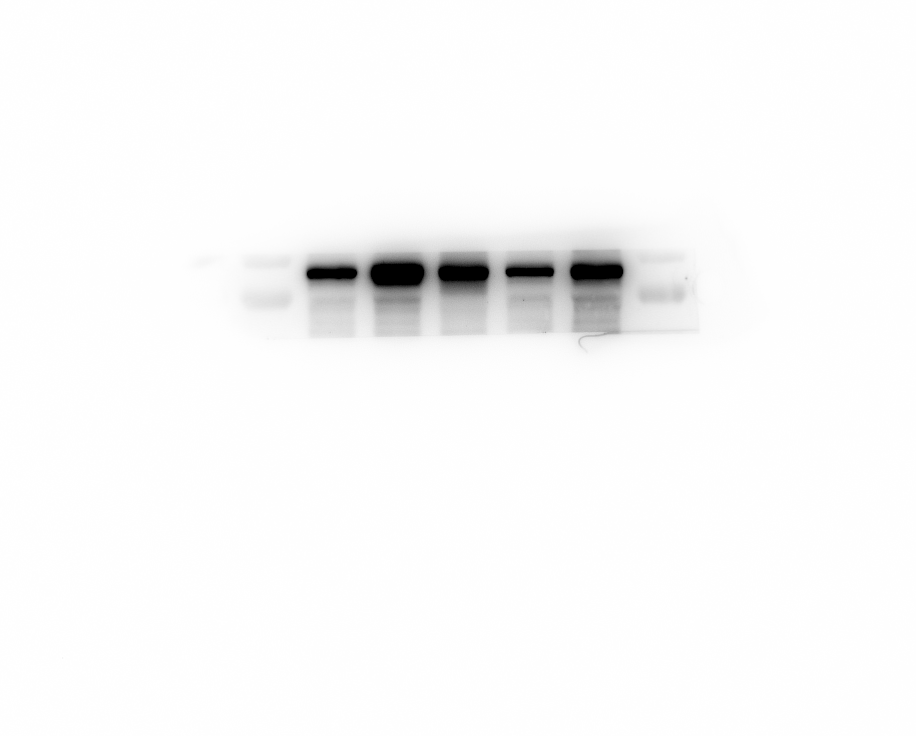

Supplement: Supplementary file 6 [file Data_Sheet_6.ZIP › WB/MMP9.tif]

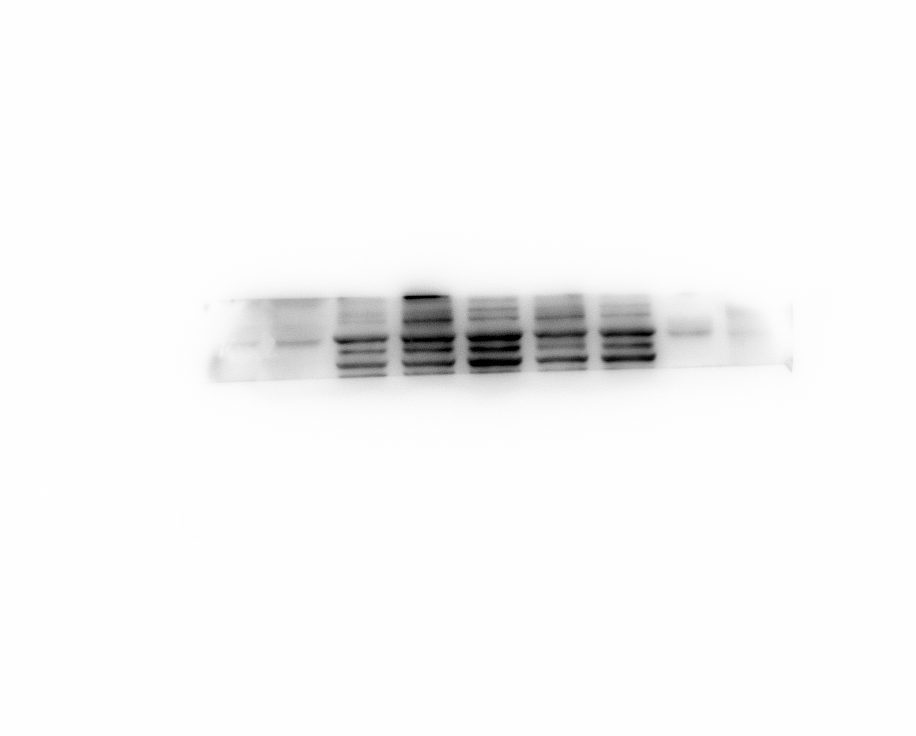

Supplement: Supplementary file 6 [file Data_Sheet_6.ZIP › WB/NOX2.tif]

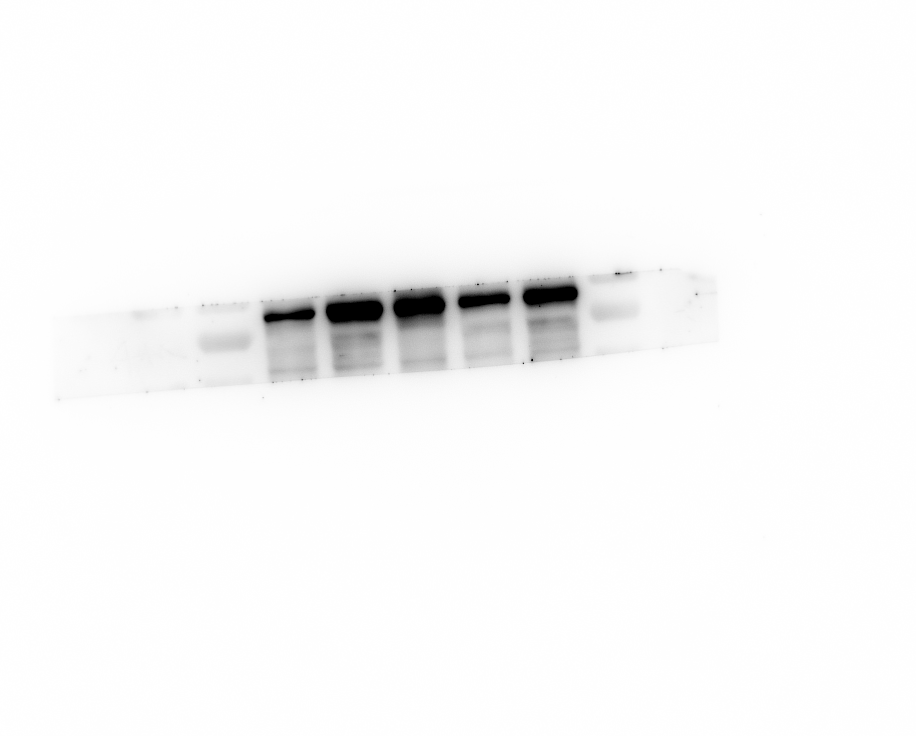

Supplement: Supplementary file 6 [file Data_Sheet_6.ZIP › WB/NOX4.tif]

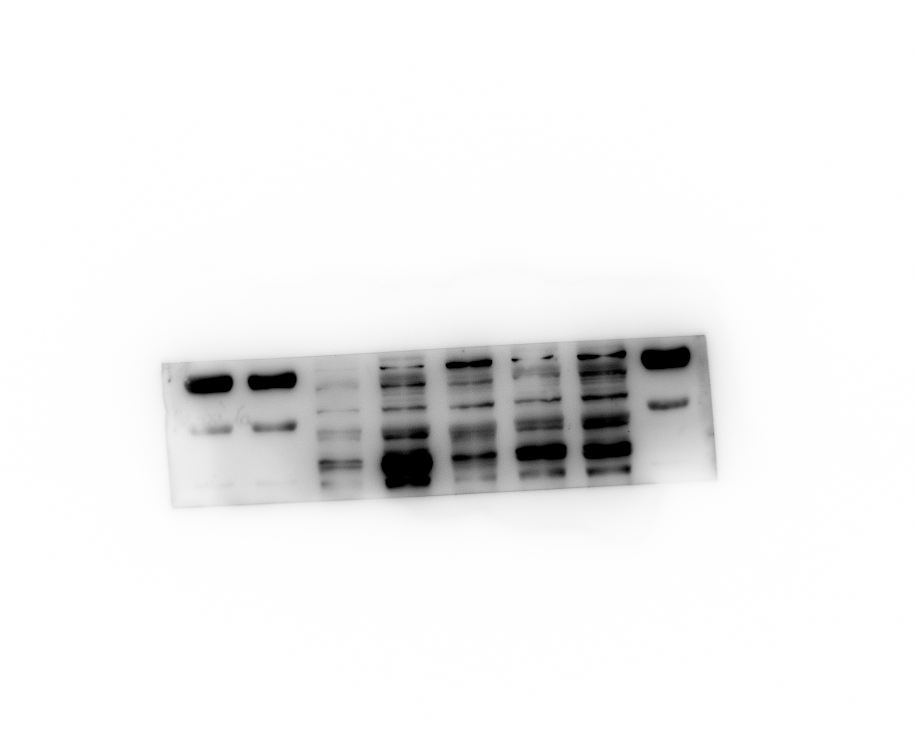

Supplement: Supplementary file 6 [file Data_Sheet_6.ZIP › WB/ox-CaMKII.tif]

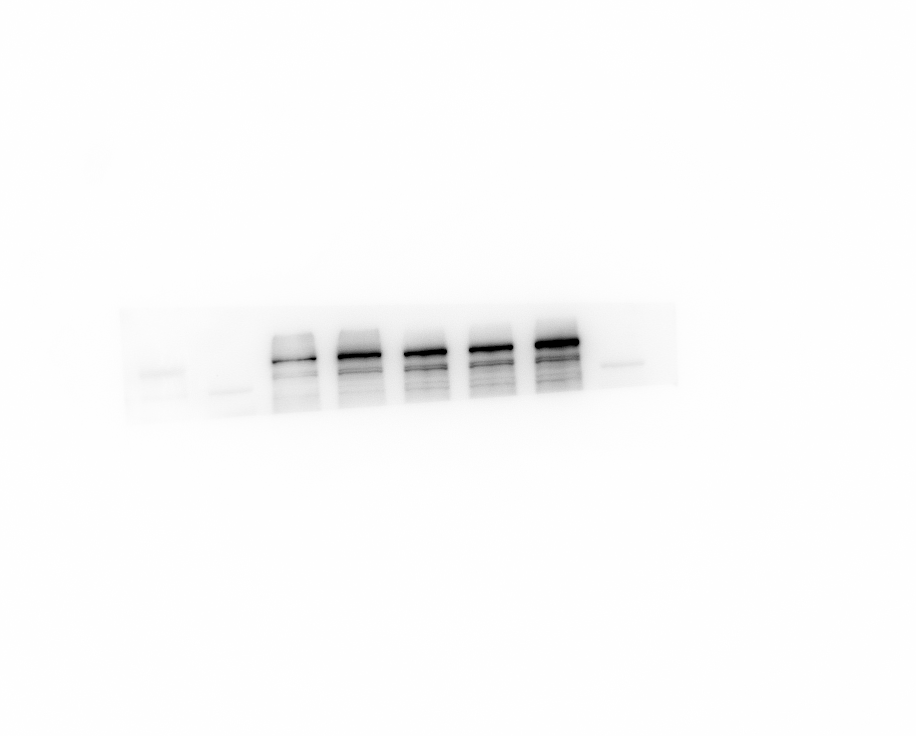

Supplement: Supplementary file 6 [file Data_Sheet_6.ZIP › WB/p-Drp1.tif]

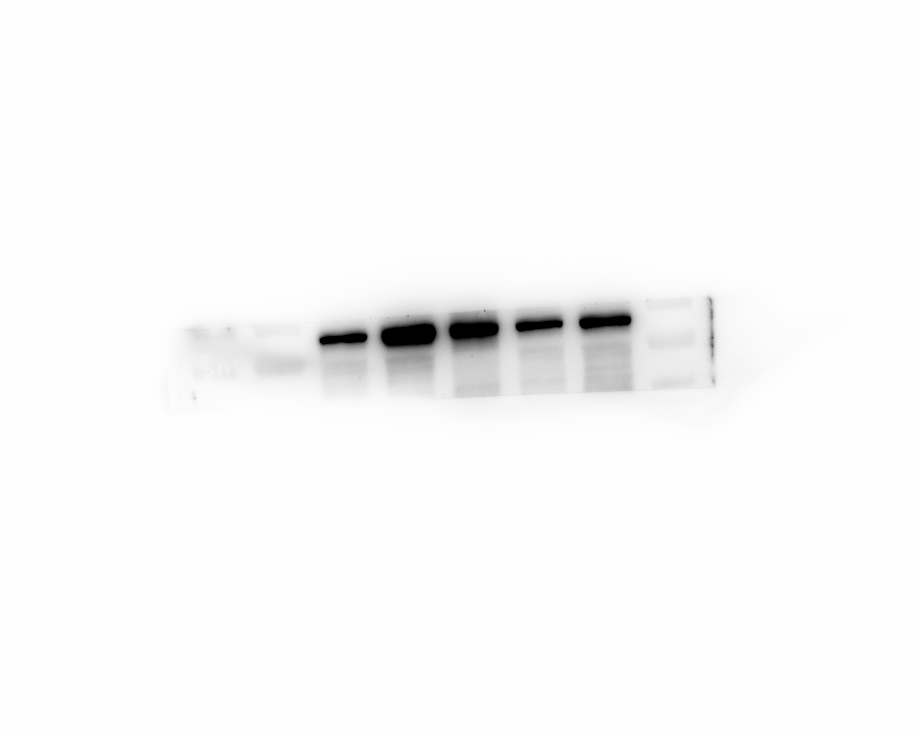

Supplement: Supplementary file 6 [file Data_Sheet_6.ZIP › WB/TGF-β.tif]

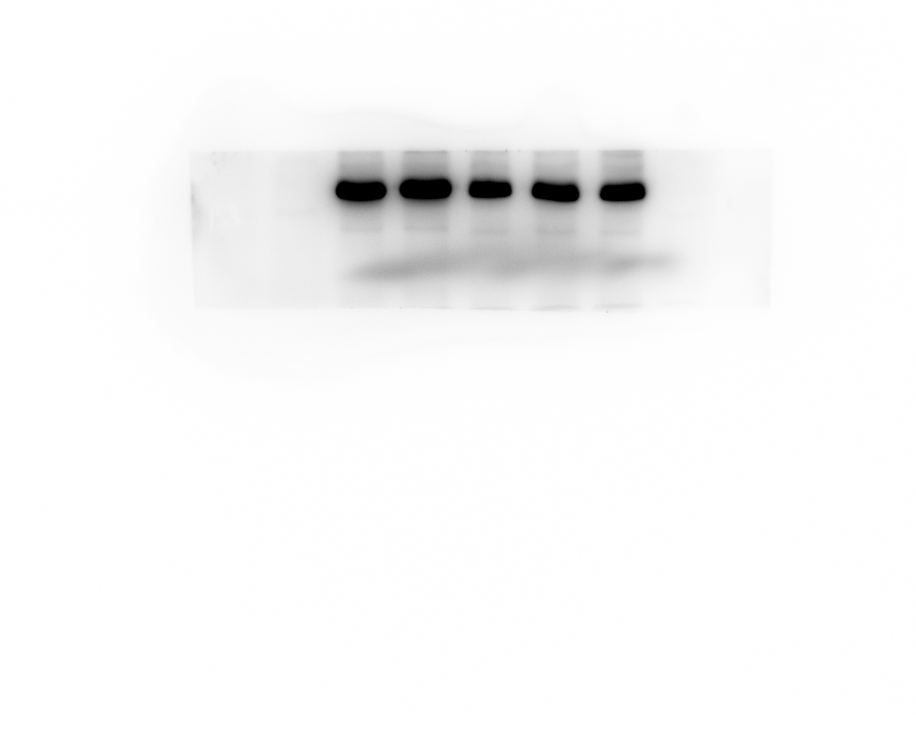

Supplement: Supplementary file 6 [file Data_Sheet_6.ZIP › WB/β-actin of CaMKII.tif]

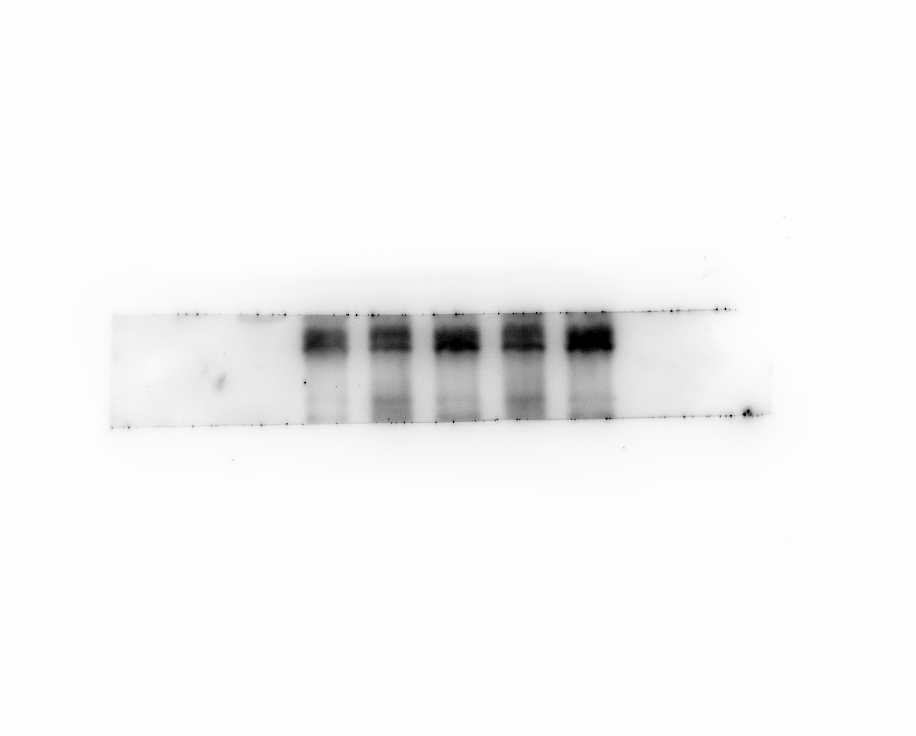

Supplement: Supplementary file 6 [file Data_Sheet_6.ZIP › WB/β-actin of CB2R.tif]

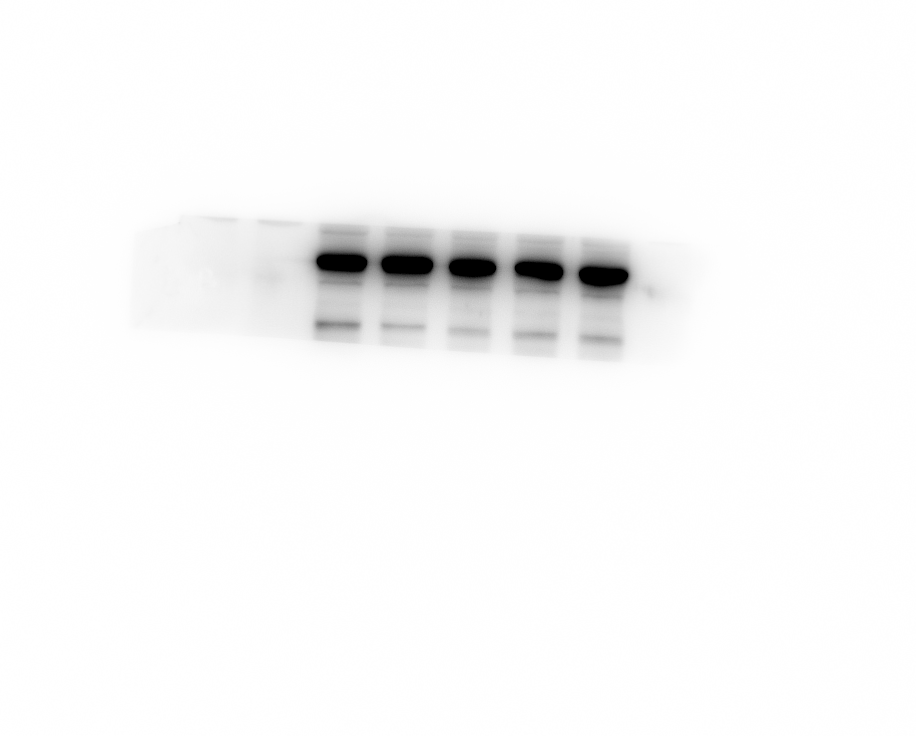

Supplement: Supplementary file 6 [file Data_Sheet_6.ZIP › WB/β-actin of Drp1.tif]

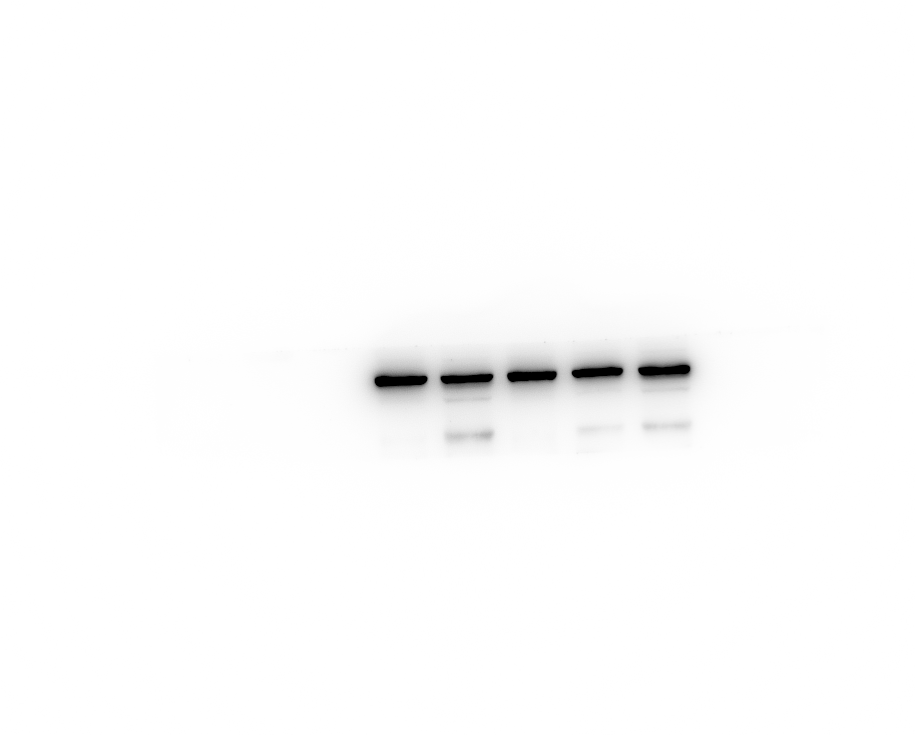

Supplement: Supplementary file 6 [file Data_Sheet_6.ZIP › WB/β-actin of fibrosis.tif]

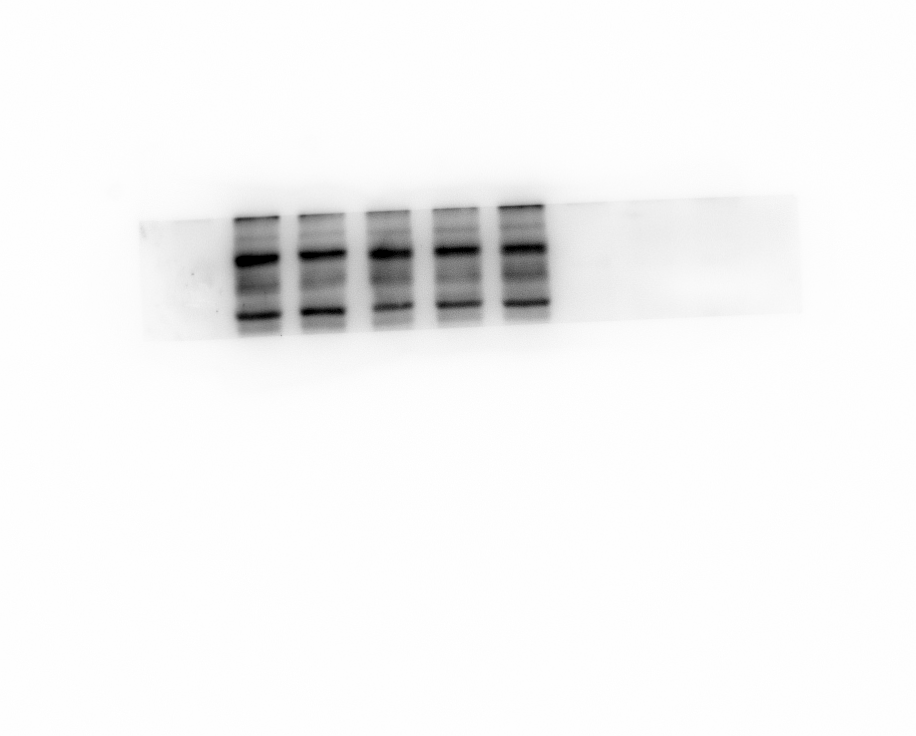

Supplement: Supplementary file 6 [file Data_Sheet_6.ZIP › WB/β-actin of NOX2.4.tif]

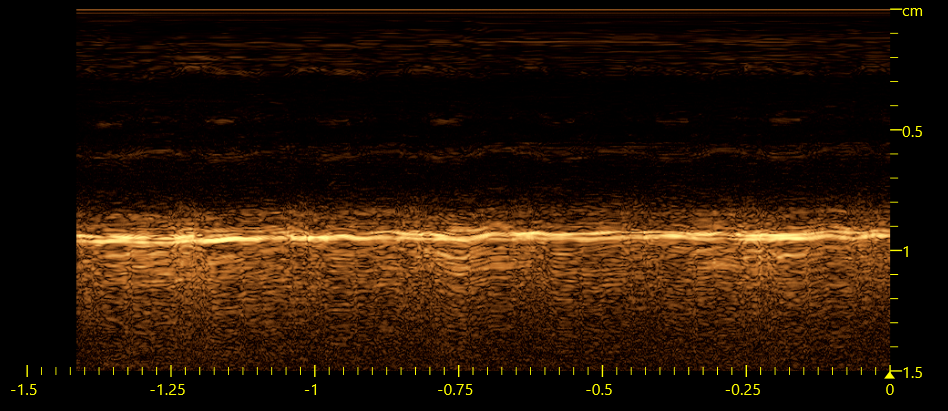

Supplement: Supplementary file 7 [file Data_Sheet_7.ZIP › LAD/Ang II.tif]

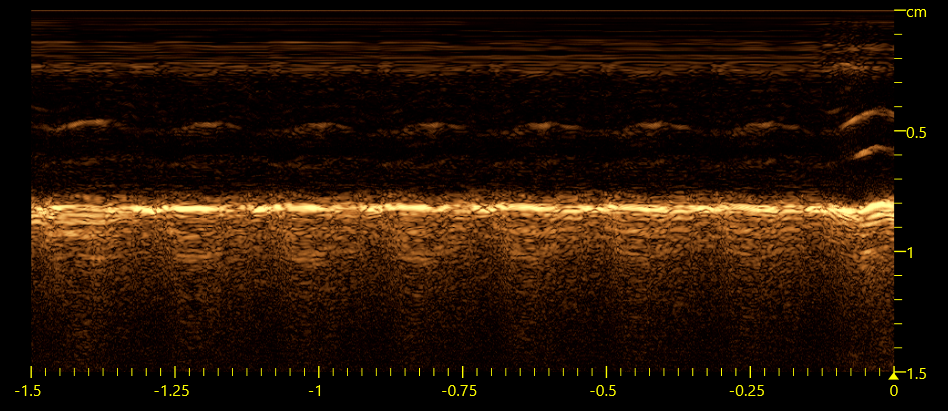

Supplement: Supplementary file 7 [file Data_Sheet_7.ZIP › LAD/Ang II+AM1241.tif]

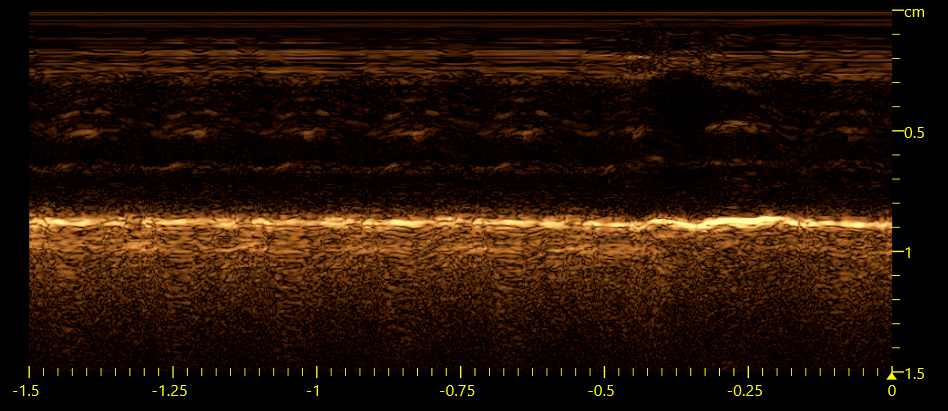

Supplement: Supplementary file 7 [file Data_Sheet_7.ZIP › LAD/Ang II+AM630.tif]

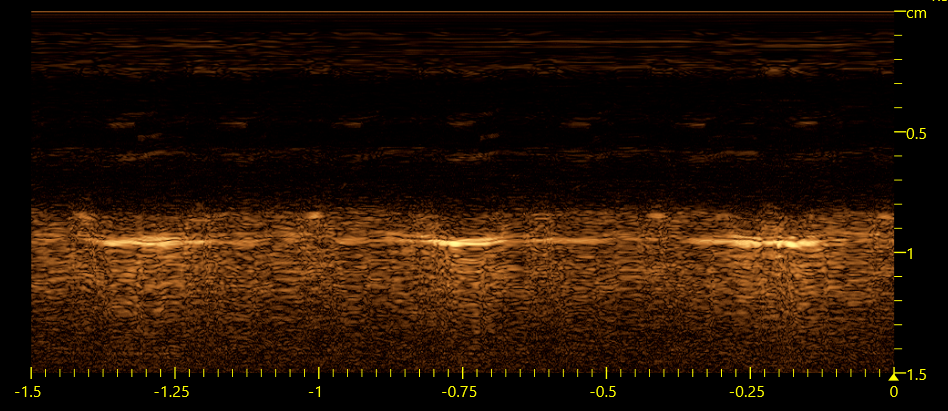

Supplement: Supplementary file 7 [file Data_Sheet_7.ZIP › LAD/Ang II+Veh.tif]

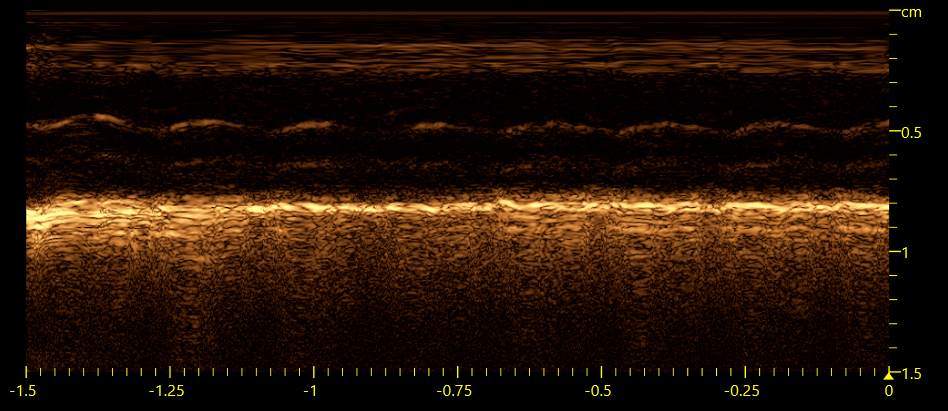

Supplement: Supplementary file 7 [file Data_Sheet_7.ZIP › LAD/Control.tif]

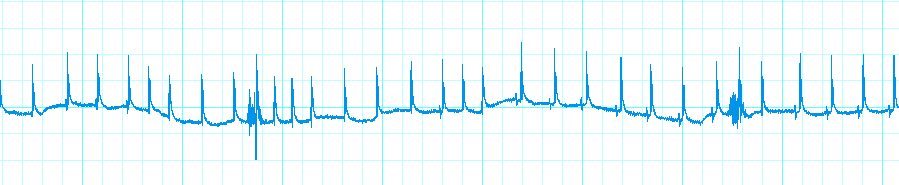

Supplement: Supplementary file 8 [file Data_Sheet_8.ZIP › AF stimulation/AF+SR.tif]

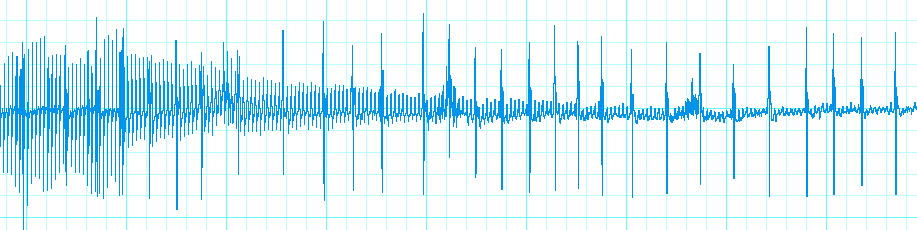

Supplement: Supplementary file 8 [file Data_Sheet_8.ZIP › AF stimulation/BURST+AF.tif]

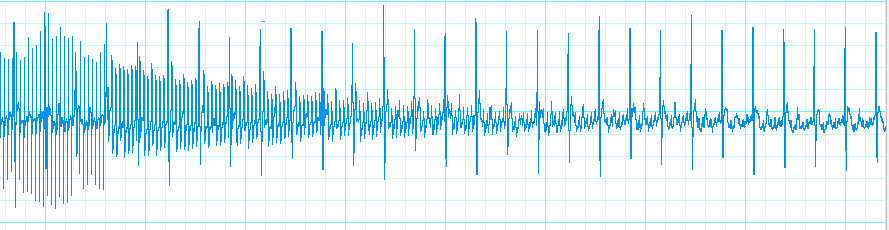

Supplement: Supplementary file 8 [file Data_Sheet_8.ZIP › AF stimulation/BURST+SR.tif]

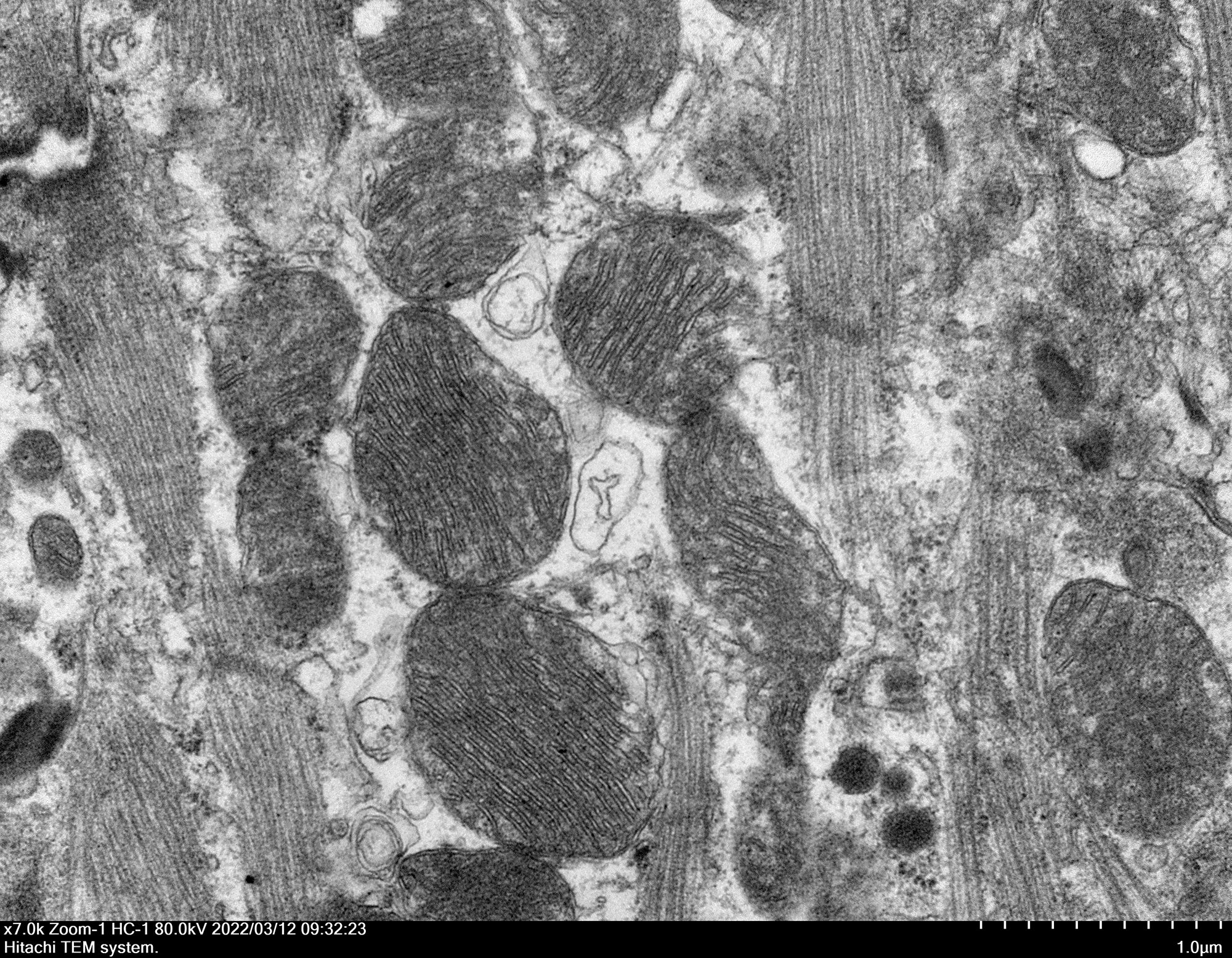

Supplement: Supplementary file 11 [file Image_1.TIF]

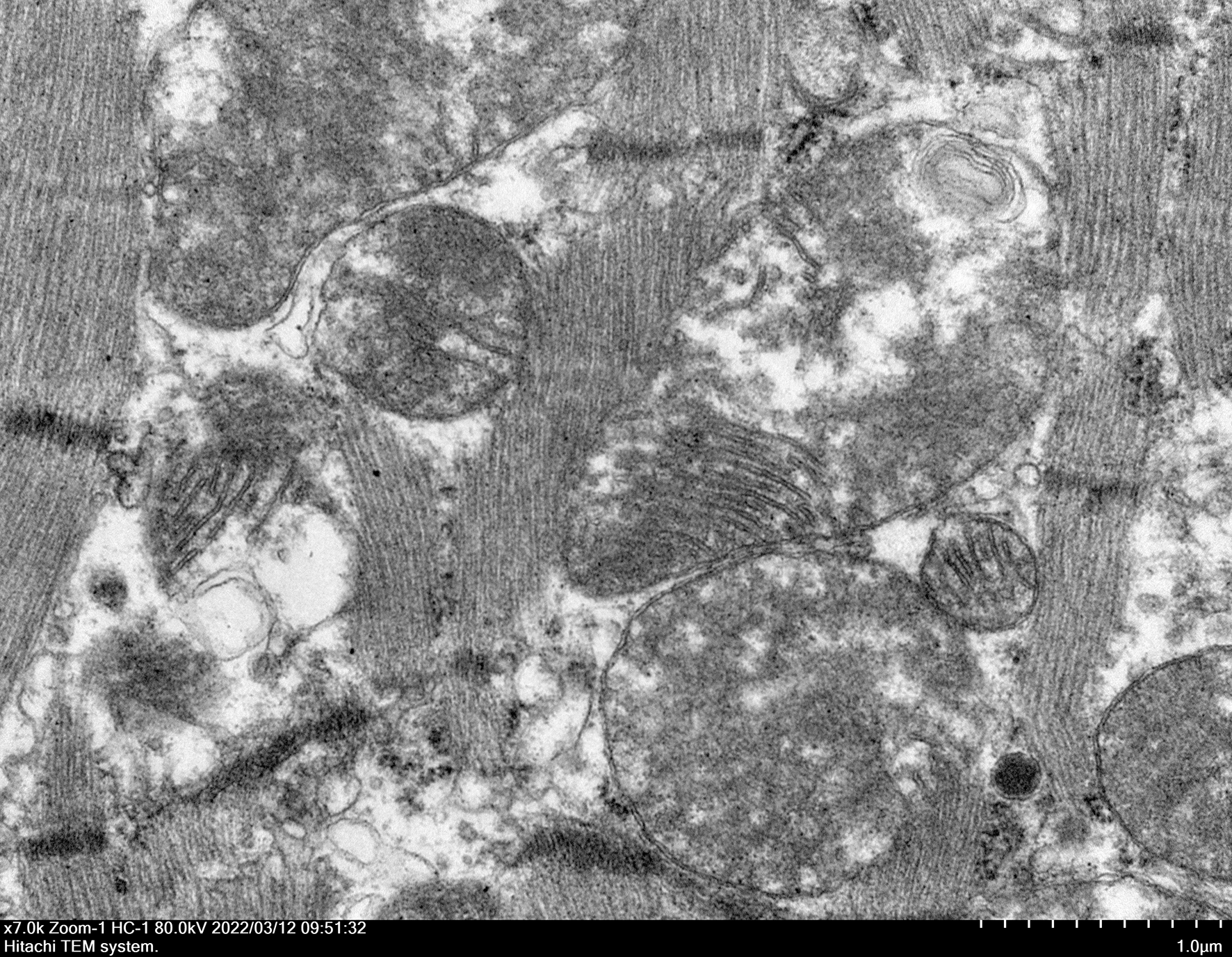

Supplement: Supplementary file 12 [file Image_2.TIF]

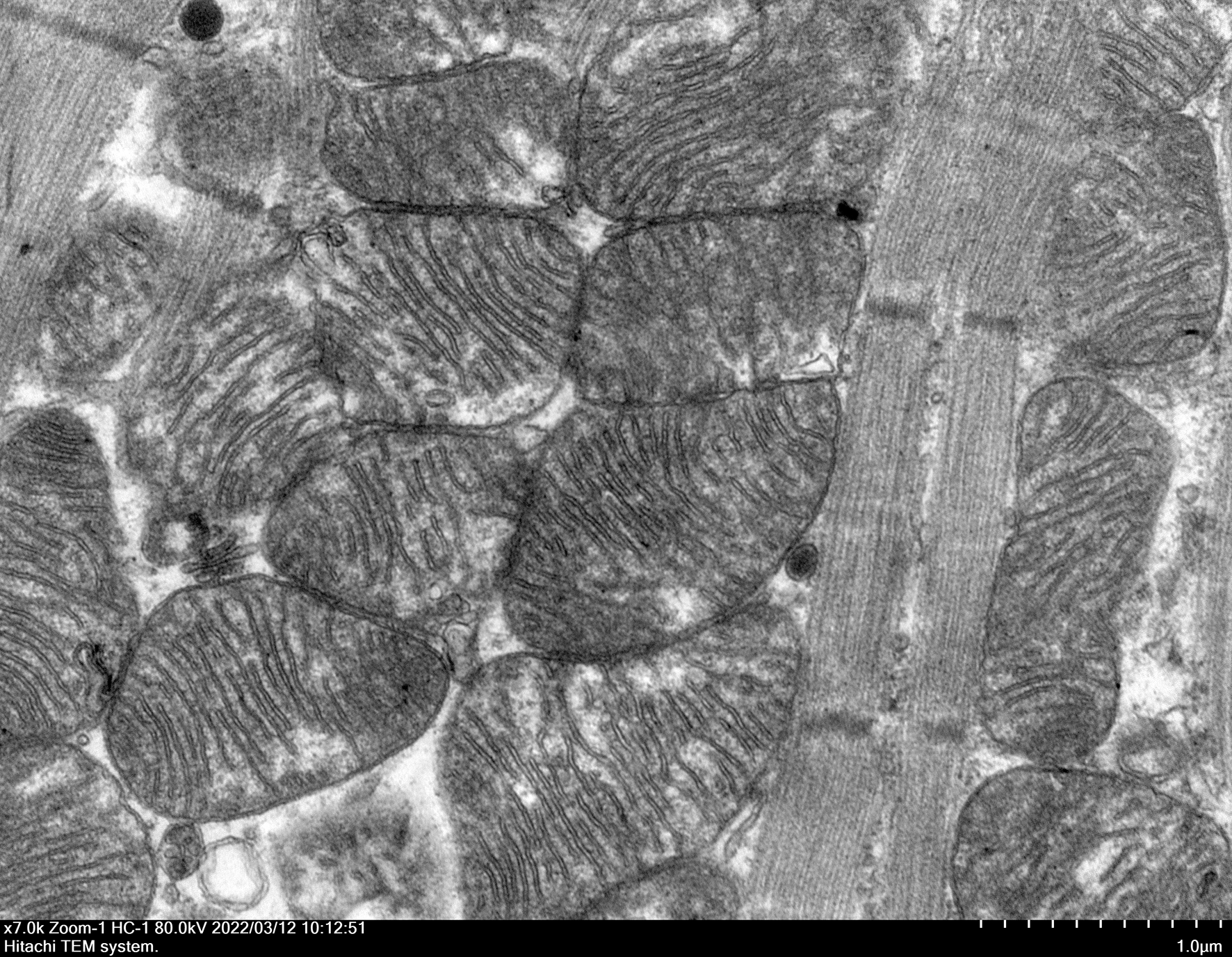

Supplement: Supplementary file 13 [file Image_3.TIF]

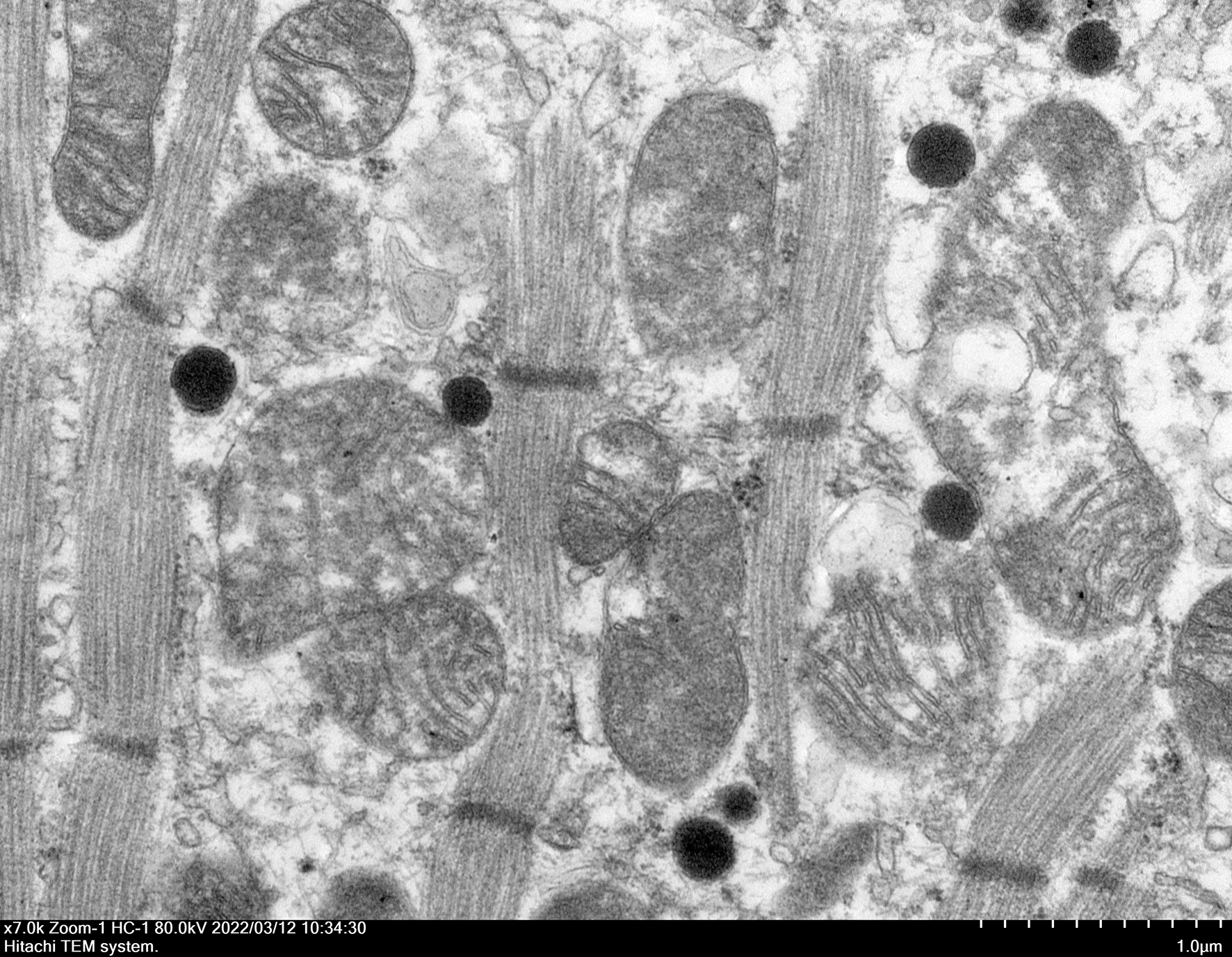

Supplement: Supplementary file 14 [file Image_4.TIF]

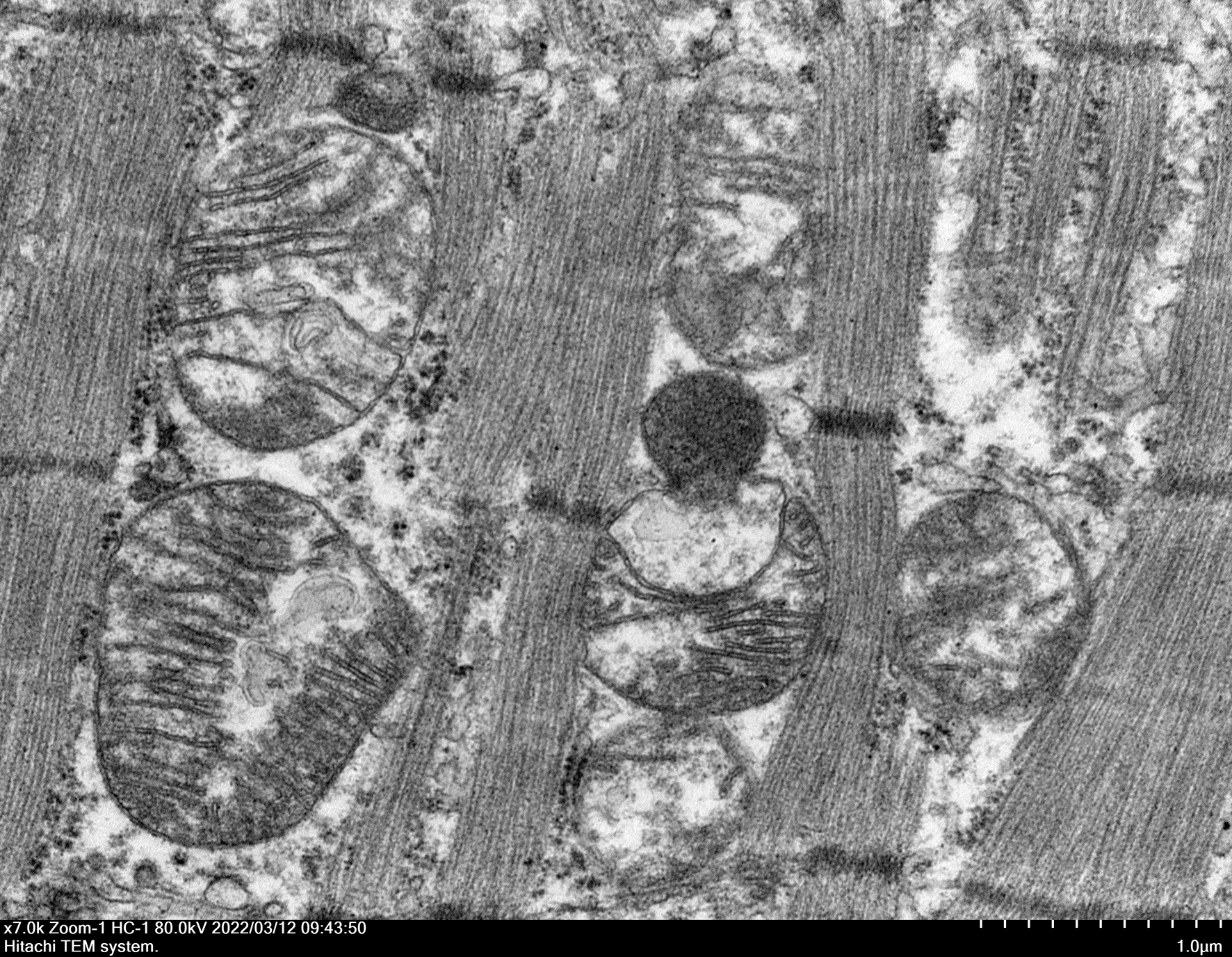

Supplement: Supplementary file 15 [file Image_5.TIF]
